# Integrated analysis of metabolome, transcriptome, and bioclimatic factors of *Acer truncatum* seeds reveals key candidate genes related to unsaturated fatty acid biosynthesis, and potentially optimal production area

**DOI:** 10.1186/s12870-024-04936-6

**Published:** 2024-04-16

**Authors:** Yang Li, Fan Kong, Shangwei Wu, Wenjin Song, Yang Shao, Min Kang, Tiantian Chen, Liping Peng, Qingyan Shu

**Affiliations:** 1 State Key Laboratory of Plant Diversity and Specialty Crops, Institute of Botany, Chinese Academy of Sciences, Beijing, 100093 China; 2 China National Botanical Garden, Beijing, 100093 China; 3 University of Chinese Academy of Sciences, Beijing, 100049 China; 4 Hunan Agricultural University, Changsha, 410128 China; 5 Taishan Academy of Forestry Sciences, Tai’an, 271002 China

**Keywords:** *Acer truncatum* seeds oil, Unsaturated fatty acids, Metabolome and transcriptome, Bioclimatic factors, Potentially optimal production area

## Abstract

**Background:**

Lipids found in plant seeds are essential for controlling seed dormancy, dispersal, and defenses against biotic and abiotic stress. Additionally, these lipids provide nutrition and energy and are therefore important to the human diet as edible oils. *Acer truncatum*, which belongs to the Aceaceae family, is widely cultivated around the world for its ornamental value. Further because its seed oil is rich in unsaturated fatty acids (UFAs)- i.e. α-linolenic acid (ALA) and nervonic acid (NA)- and because it has been validated as a new food resource in China, the importance of *A. truncatum* has greatly risen. However, it remains unknown how UFAs are biosynthesized during the growth season, to what extent environmental factors impact their content, and what areas are potentially optimal for their production.

**Results:**

In this study, transcriptome and metabolome of *A. truncatum* seeds at three representative developmental stages was used to find the accumulation patterns of all major FAs. Cumulatively, 966 metabolites and 87,343 unigenes were detected; the differential expressed unigenes and metabolites were compared between stages as follows: stage 1 vs. 2, stage 1 vs. 3, and stage 2 vs. 3 seeds, respectively. Moreover, 13 *fatty acid desaturases* (*FADs*) and 20 *β-ketoacyl-CoA synthases* (*KCSs*) were identified, among which the expression level of *FAD3* (*Cluster-7222.41455*) and *KCS20* (*Cluster-7222.40643*) were consistent with the metabolic results of ALA and NA, respectively. Upon analysis of the geographical origin-affected diversity from 17 various locations, we found significant variation in phenotypes and UFA content. Notably, in this study we found that 7 bioclimatic variables showed considerable influence on FAs contents in *A. truncatum* seeds oil, suggesting their significance as critical environmental parameters. Ultimately, we developed a model for potentially ecological suitable regions in China.

**Conclusion:**

This study provides a comprehensive understanding of the relationship between metabolome and transcriptome in *A. truncatum* at various developmental stages of seeds and a new strategy to enhance seed FA content, especially ALA and NA. This is particularly significant in meeting the increasing demands for high-quality edible oil for human consumption. The study offers a scientific basis for *A. truncatum*’s novel utilization as a woody vegetable oil rather than an ornamental plant, potentially expanding its cultivation worldwide.

**Supplementary Information:**

The online version contains supplementary material available at 10.1186/s12870-024-04936-6.

## Background

*Acer truncatum*, which belongs to the *Acer* genus in the Aceaceae family, is a woody tree species native to northern China, Japan, and Korea. Today, it is cultivated globally for its ornamental and oil value [1]. Furthermore, up to 90% of the seed oil content consists of unsaturated fatty acids (UFAs) including 25.19% oleic acid (C18:1, OA), 32.97% linoleic acid (C18:2, LA), 2.76% α-linolenic acid (C18:3, ALA), 7.90% eicosenoic acid (C20:1, ESA), 16.49% erucic acid (C22:1, EA), and 5.76% nervonic acid (C24:1, NA) [2, 3]. ALA, a polyunsaturated fatty acid, is an essential human dietary nutrient, but it cannot be synthesized independently by the human body [4]. NA, a long-chain monounsaturated fatty acid, is a core component of the brain’s nervous system and has a healing effect on damaged nerve fibers [5]. NA cannot be synthesized by the human body either. Historically, NA has been obtained through shark brains. However, due to a shark hunting ban, obtaining NA through plant sources has become a matter of interest [5]. Compared to sources from animals, the plant sources of NA present numerous advantages, including cost-effectiveness and high concentration. Further, plants can be cultivated in batches, which enables efficient extraction of NA at a much larger scale. In 2011, the Ministry of Health of China approved *A. truncatum* seeds oil (ATSO) as a new food resource due to its high nutritional value and rich content of UFAs [6]. As an important plant source of NA, the seed oil of *A. truncatum* has drawn widespread attention [7]. Currently, several studies have been carried out on the oil content, fatty acid composition, extraction technology, etc. regarding as ATSO. However, systematic exploration of the relationship between transcriptome and metabolome of the developmental seeds of *A. truncatum* and optimal production area predictions are still insufficient. Ultimately, this negatively affects the production industry of ATSO and UFAs, especially the supply of NA.

Currently, the biosynthetic pathway of UFAs is well understood. Firstly, UFAs are synthesized *de novo* in plastids, which begins from pyruvate and is then catalyzed by various enzymes: pyruvate dehydrogenase complex (PDHC), acetyl-CoA carboxylase (ACCase), malonyl-CoA-ACP transacylase (MCAAT), β-ketoacyl-ACP synthase III (KAS III), β-ketoacyl-ACP reductase (KAR), β-hydroxyacyl-ACP dehydrase (HAD) and enoyl-ACP reductase (EAR) in turn to form C4:0-ACP. Following the catalytic condensation by KASI, C4:0-ACP enters the cycle again and produces C6-C16 fatty acids through numerous cycles. The saturated fatty acid C16:0-ACP is finally condensed into C18:0-ACP by KASII. C18:0-ACP is then desaturated by stearoyl-ACP desaturase (SAD), which is the first desaturation step to produce C18:1-ACP. The subsequent desaturation process is carried out by the important enzymes of fatty acid desaturases (FADs). These FADs are majorly divided into ω-6 FADs (including FAD2 and FAD6) and ω-3 FADs (including FAD3, FAD7, and FAD8). In the plastid, C18:1-ACP is desaturated by FAD6 to produce C18:2-ACP. C18:3-ACP is then obtained by FAD7/8. Finally, ACP is removed by acyl-ACP thioesterase A/B (FATA/B) or palmitoyl-CoA hydrolase (PCH) to produce free palmitic acid (C16:0, PA), stearic acid (C18:0, SA), OA, LA and ALA. FAD4 and FAD5 act on PA to produce palmitoleic acid (C16:1) in plastids, which are specifically from phosphatidylglycerol and monogalactosyldiacylglycerol, respectively [8–12]. At present, FADs have been identified in many oil crops, including *Glycine max* [13], *Brassica napus* [14], and *Olea europaea* [15]. The study on FAD in *A. truncatum* recently reported, six putative *FADs* were clustered into five *FAD* groups in its transcriptome. However, there are only few reports on FADs in *A. truncatum*, and in-depth and comprehensive studies are still needed to fully understand their function on UFA biosynthesis.

In some higher plants, very long chain fatty acid (VLCFA) is obtained by carbon chain lengthening reactions using OA as a substrate, which further lengthens the carbon chain in the endoplasmic reticulum (ER). The cycle is divided into four steps: in the first step, fatty acyl-CoA and malonyl-CoA are catalyzed by β-ketoacyl-CoA synthase (KCS) to produce β-ketoacyl-CoA with two additional carbons. KCS is a key rate-regulating enzyme for the elongation of fatty acid carbon chains. Then, β-ketoacyl-CoA reductase (KCR) catalyzes the reduction reaction of β-ketoacyl-CoA to produce β-hydroxyacyl-CoA. In the third step, the dehydration reaction of β-hydroxyacyl-CoA is catalyzed by β-hydroxyacyl-CoA dehydrase (HCD) to produce enoyl-CoA. Finally, enoyl-CoA reductase (ECR) catalyzes the reduction of enoyl-CoA to produce a long-chain fatty acyl-CoA with two added carbons to the beginning products. This then enters to the cycle again. Through the elongation of the carbon chain, VLCFAs such as ESA, EA, and NA are eventually produced [16, 17]. *KCSs* were first identified in *Arabidopsis thaliana*, containing 21 *KCSs*, among which *AtKCS18* (*At4g34520*) was the first cloned one and it extends the chain length of fatty acids from C18 to C20 and C22 [18]. In recent years, KCSs have been reported in other species, including *Helianthus annuus* [19] and six *Citrinae* species [20]. In *A. truncatum*, Ma et al. [17] identified 28 putative *KCSs*. However, the relationship between the transcriptional difference of *KCS* and the metabolites accumulation during *A. truncatum* seed development is not well understood.

The content of seed fatty acids is always affected by a lot of bioclimatic factors. Different origins of *A. truncatum* may result in different qualities of ATSO as well as its products in diet. It thrives in fruitful soil but can grow in acidic, neutral or alkaline soil [21] with its developed root system providing environmental adaptation. The morphology, growth condition, and the content of seed FAs of *A. truncatum* vary with the plant’s geographic environment and bioclimatic changes [22]. Recent research showed that the oil yields of *A. truncatum* ranged from 7.27 to 35.05% depending on various geographic locations [23], while the leaf length decreased considerably with increased annual average temperature and annual precipitation. The choice of appropriate cultivation area has significant consequences for *A. truncatum*’s sustainable use since climate, terrain, and soils of various regions affect the growth and seed quality. Therefore, for the introduction and cultivation of this plant in China, it is necessary to map the climatically suitable producing areas for *A. truncatum* and determine critical environmental parameters.

In this study, we firstly monitored the accumulation pattern of UFAs during seed development of *A. truncatum* using gas chromatography–mass spectrometry (GC-MS) and identified three key stages of FAs accumulation for subsequent omics analysis. We aimed to investigate the dynamic changes in metabolites, especially FAs, and identify relevant regulatory genes in developing *A. truncatum* seed at three key stages by integrative analysis of metabolome and transcriptome. In order to understand the effects of environmental factors on seed phenotype and FA content, we evaluated regional differences of morphological characteristics and FAs content in the seeds from 17 main production areas and analyzed the influences of 7 selected bioclimatic factors on FAs content in seeds. Finally, we used the MaxEnt model based on the distribution of data from 116 distinct locations and seven bioclimatic variables to predict environmentally suitable production areas for *A. truncatum.* In addition, this study investigated the differentiation degree of phenotypic traits among various populations and the relationship between phenotypic traits variation and environmental factors to provide a scientific basis for further development and utilization of the germplasm resources of *A. truncatum* and the optimal production area for high quality of ATSO.

## Results

### Metabolic diversity during *A. truncatum* seeds development

**Fig. 1:**
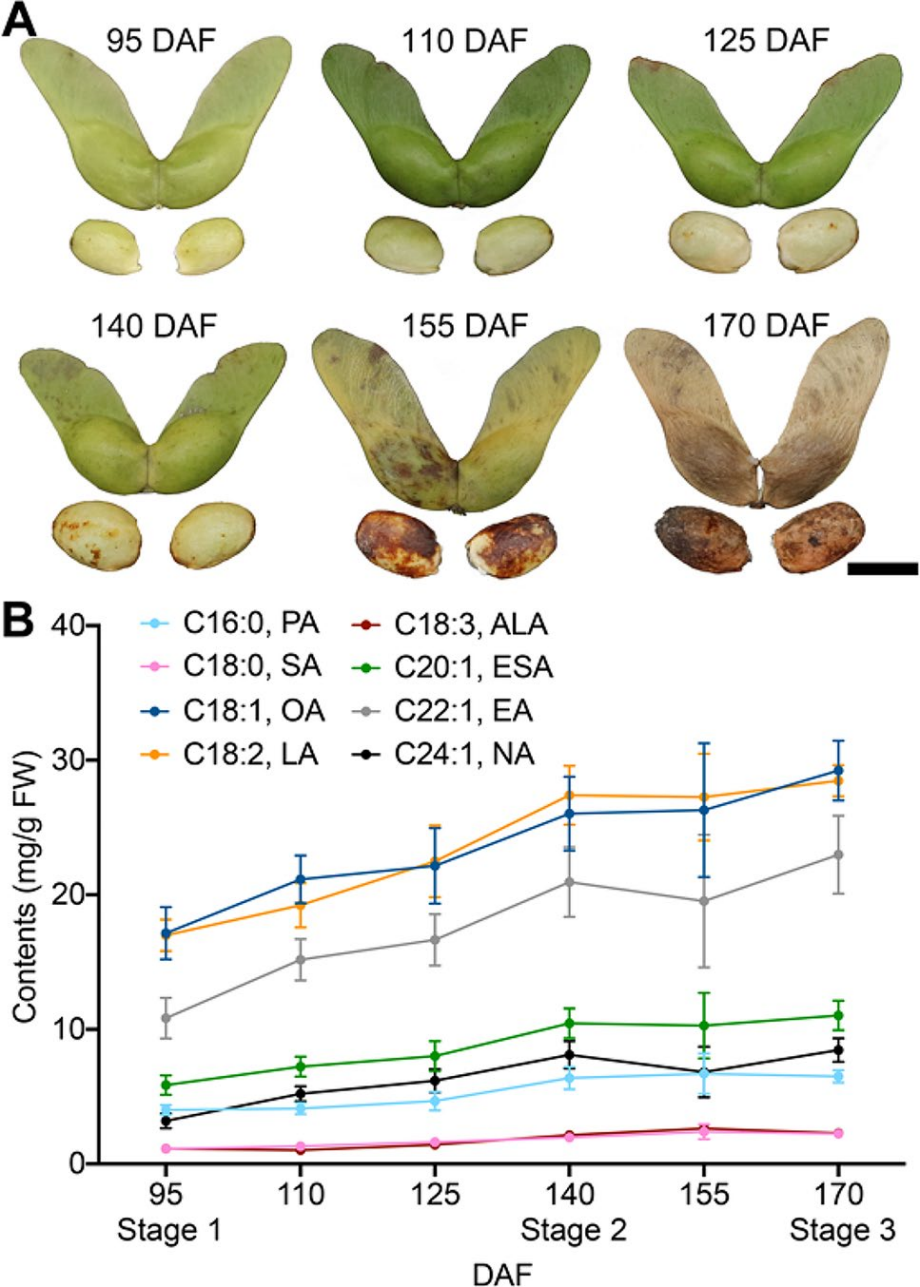
The FAs accumulation pattern of *A. truncatum* seeds at six time points during their development. (**A**) The developmental time points of *A. truncatum* seed. Six time points during *A. truncatum* seeds development were selected: 95, 110, 125, 140, 155, and 170 DAF. (**B**) Content of accumulation pattern of eight main FAs in *A. truncatum* seed using GC-MS during seed development. The mean values ± SD from three biological replicates (*n* = 3) are shown. PA, palmitic acid; SA, stearic acid; OA, oleic acid; LA, linoleic acid; ALA, α-linolenic acid; ESA, eicosenoic acid; EA, erucic acid; NA, nervonic acid. DAF, days after flowering

To test the FAs accumulation pattern during *A. truncatum* seed development, seeds at 95, 110, 125, 140, 155, and 170 days after flowering (DAF) were selected for oil content and composition measurements (Fig. 1A). A total of eight major FAs were obtained using GC-MS analysis: PA, SA, OA, LA, ALA, ESA, EA, and NA. During seed development, the total FAs content increased from 95 DAF (60.29 mg/g FW) to 140 DAF (103.45 mg/g FW) rapidly, increased slightly from 140 DAF to 170 DAF (111.22 mg/g FW), and reached the highest content at 170 DAF (Fig. 1B). Among UFAs, the contents of OA, LA, ALA, ESA, EA and NA rapidly increased from 95 DAF to 140 DAF, which were from 17.15 to 26.04, 16.99 to 27.40, 1.13 to 2.15, 5.86 to 10.45, 10.84 to 20.96 and 3.19 to 8.11 mg/g FW and slightly increased from 140 DAF to 170 DAF (29.23, 28.48, 2.29, 11.03, 22.98, and 8.46 mg/g FW), respectively. Based on the above results, we selected 95 (stage 1), 140 (stage 2) and 170 DAF (stage 3) as three key stages for subsequent omics analysis.

In order to further understand the diversity of metabolite accumulation during seed development of *A. truncatum*, we conducted widely targeted metabolomic analysis from stage 1–3 seeds. A total of 966 metabolites were detected, including 40 alkaloids, 92 amino acids and derivatives, 217 flavonoids, 43 lignans and coumarins, 135 lipids, 54 nucleotides and derivatives, 78 organic acids, 164 phenolic acids, 23 tannins, 15 terpenoids, and 105 others (Fig. S1). Principal component analysis (PCA) results showed that the first and second principal components can explain 50.8% and 28.3% of the total variance, respectively, and they can distinctly distinguish stage 1–3 seed sample (Fig. S2A). Differential metabolites (DMs) between groups were selected by VIP ≥ 1 and absolute log_2_ fold change ≥ 1. There were 334 (84 up- and 250 down-regulated), 441 (213 up- and 228 down-regulated) and 314 (244 up- and 70 down-regulated) DMs being compared in stage 1 vs. 2, stage 1 vs. 3, and stage 2 vs. 3 seeds, respectively (Table S1). The results of kyoto encyclopedia of genes and genomes (KEGG) pathway enrichment showed that DMs were all enriched in purine metabolism and linoleic acid metabolism in the above compared groups. Moreover, DMs were both enriched in ALA metabolism in stage 1 vs. 3 and stage 2 vs. 3 (Fig. S3).

### Transcriptome analysis of the developing *A. truncatum* seeds

To further explore the gene expression changes involved in the biosynthesis of FAs during seed development, the transcriptome analysis was carried out at three developmental stages. The average raw and clean reads were 46,844,390 and 45,117,354, respectively. The clean Q30 base rate was higher than 94.58%, and the mapped ratio was 83.74% from transcriptome, which indicated the high-quality sequences for further analysis (Table S2). In total, 87,343 unigenes, with an average length of 1,022 bp, were obtained after filtering out low quality reads. PCA results showed that the first and second principal components can explain 32.4% and 17.1% of the total variance, respectively, and they can distinguish stage 1–3 seeds samples clearly (Fig. S2B). Differential expression genes (DEGs) between compared groups were selected by FDR < 1 and absolute log_2_ fold change ≥ 1. There were 6,846 (2,868 up- and 3,978 down-regulated), 16,129 (7,428 up- and 8,701 down-regulated) and 11,937 (5,997 up- and 5,940 down-regulated) DEGs in stage 1 vs. 2, stage 1 vs. 3, and stage 2 vs. 3 seeds, respectively (Table S1). The results of KEGG pathway enrichment showed that DEGs were all enriched in plant hormone signal transduction, phenylpropanoid biosynthesis, metabolic pathways, flavonoid biosynthesis, fatty acid metabolism and biosynthesis of secondary metabolites in the above comparison group. Moreover, DEGs were enriched in fatty acid degradation, fatty acid biosynthesis and biosynthesis of unsaturated fatty acids in stage 1 vs. 2 and stage 2 vs. 3 seeds (Fig. S4), which suggests that important changes in genes related to fatty acids regulation occurred during seed development of *A. truncatum*.

### Key enzyme genes of *FAD* and *KCS* families indicated various expression profile in *A. truncatum* during seed development

**Fig. 2:**
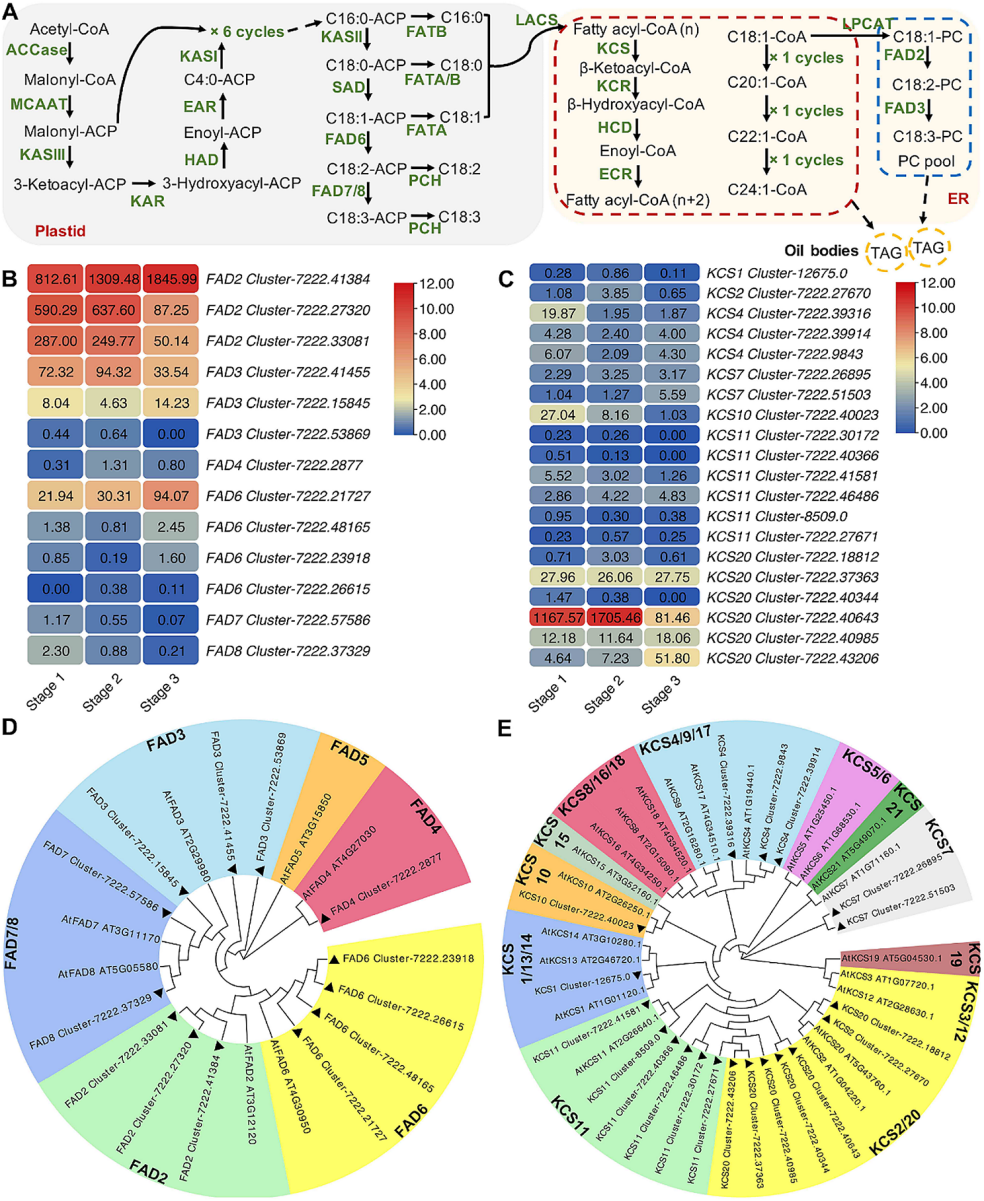
Transcriptomic profiles of genes putatively involved in the FAs biosynthetic pathway. (**A**) Sketch of FAs biosynthetic pathway. Acetyl-CoA undergoes a series of reactions to produce C18:0-ACP, followed by desaturation to produce C18:1, C18:2, and C18:3. C18:1 can be further extended to produce VLCFA. Copyright permission of KEGG pathway maps (map00061) was obtained from Kanehisa laboratory by using the copyright permission request form in www.kegg.jp/kegg/kegg1.html. (**B** & **C**) Expression of (**B**) *FADs* and (**C**) *KCSs* involved in the FAs biosynthesis. The scales refer to log_2_FPKM. (**D** & **E**) The phylogenetic trees of (**D**) AtruFADs and AtFADs, and (**E**) AtruKCSs and AtKCSs. The phylogenetic trees of AtruFADs and AtFADs, and AtruKCSs and AtKCSs were constructed using the Maximum Likelihood method based on WAG + G and LG + G + I model respectively, within MEGA software (version X) and Evolview (http://www.evolgenius.info/evolview.html). The triangle marks the protein from *A. truncatum*

Acetyl-CoA undergoes a series of reactions to produce C18:0-ACP, followed by desaturation to produce C18:1, C18:2 and C18:3, among which FADs played an important role [12, 31]. OA can be further extended to produce VLCFA through the action of KCS, KCR, HCD and ECR in turn. Among them, KCS is a key rate-limiting enzyme that determines the tissue and substrate specificity for fatty acid elongation (Fig. 2A).

In the transcriptome, we screened 13 *FADs*, including 3 *FAD2*, *3 FAD3*, *1 FAD4*, *4 FAD6*, *1 FAD7*, and *1 FAD8* (Fig. 2B & D; Table S3). Among these *FAD*s, *FAD2* had the highest expression levels, especially *Cluster-7222.41384*, whose FPKM increased during seed development and up to 1845.99 at stage 3. While the other *FAD2* (*Cluster-7222.27320* and *Cluster-7222.33081*) were both highly expressed at stage 1–2, they decreased at stage 3, which is consistent with the metabolic result finding LA (C18:2) increased rapidly at stage 1–2 and slowly at stage 2–3 (Figs. 1B and 2B). Moreover, the expression level of *FAD6* (*Cluster-7222.21727*) also increased during seed development and reached the highest level at stage 3. Compared with *ω6-FAD*s, *ω3-FAD*s had differing expression patterns. Generally, *FAD3* had higher expression levels than that of *FAD7*/*8*, in which the transcript of *Cluster-7222.41455* increased from stage 1 to 2, but decreased at stage 3, which is consistent with the metabolic result finding that ALA (C18:3) raised rapidly at stage 1–2 and slowly at stage 2–3. The expression level of another *FAD3* (*Cluster-7222.15845*) was decreased from stage 1 to 2 but increased to the highest at stage 3. However, the *ω3-FAD*s in plastid (*FAD7*/*8*) had low expression levels during seed development and reached the highest at stage 1 and gradually decreased with seed development (Fig. 2B).

A total of 20 *KCSs* were identified from the transcriptome, including 1 *KCS1*, 1 *KCS2*, 3 *KCS4*, 2 *KCS7*, 1 *KCS10*, 6 *KCS11* and 6 *KCS20* (Fig. 2C & E, Table S4). Among these *KCS*s, *KCS20* (*Cluster-7222.40643*) had the highest expression levels, which was highly expressed at stage 1–2, but decreased sharply at stage 3. This is consistent with the metabolic result that found ESA (C20:1), EA (C22:1) and NA (C24:1) raised rapidly at stage 1–2 and slowly at stage 2–3 (Figs. 1B and 2C). The results suggested that *KCS20* (*Cluster-7222.40643*) is critically important in VLCFA metabolism in *A. truncatum*. Moreover, the expression pattern of four selected genes determined by quantitative RT-PCR (RT-qPCR) was similar to the RNA-seq results, thus indicating its reliability (Fig. S5).

### DEGs demonstrated high correlation with DMs during seed development

**Fig. 3:**
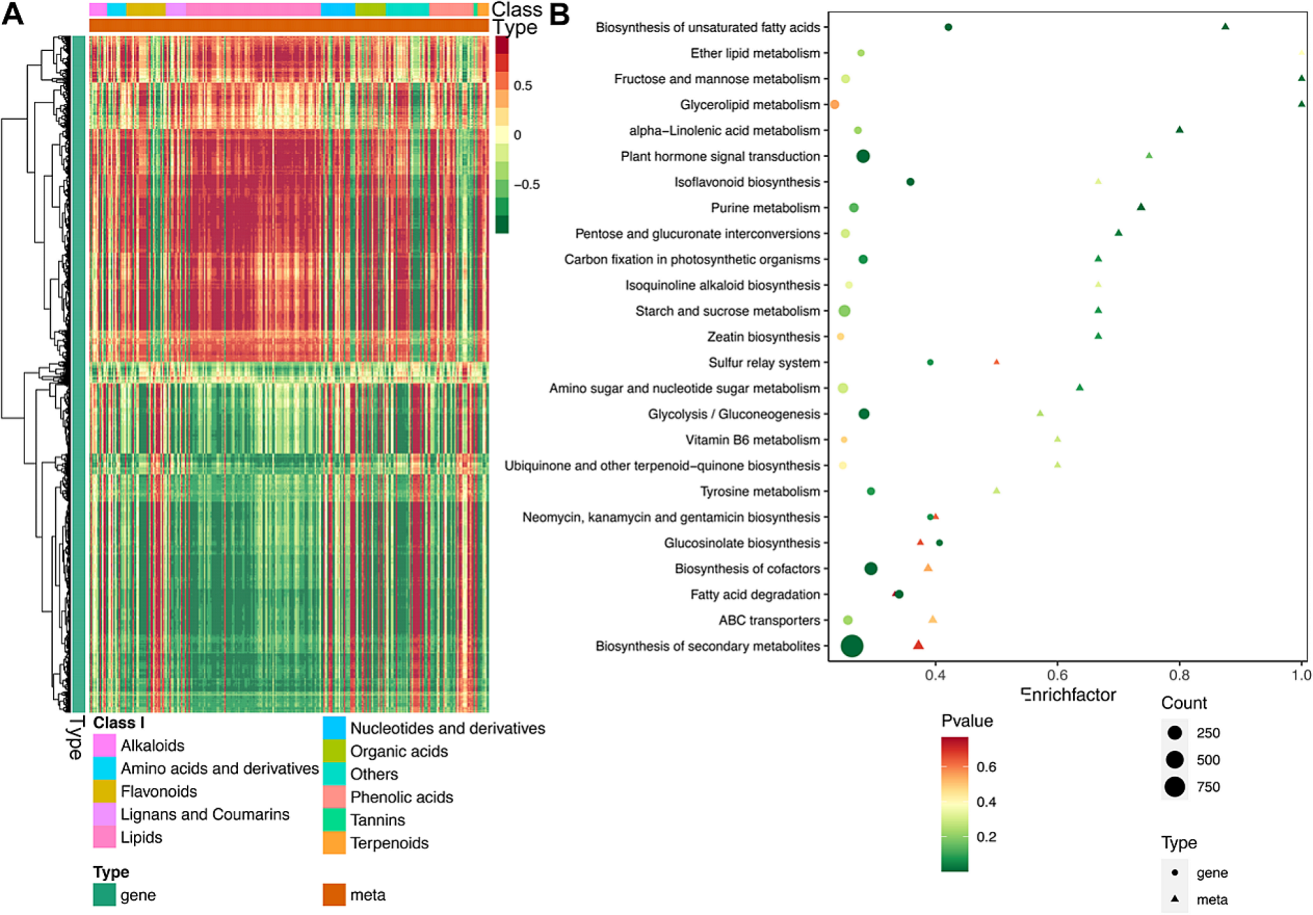
Correlation and KEGG pathways analysis of DMs and DEGs in stage 2 vs. 3 of *A. truncatum* seed. (**A**) Correlation heatmap of all the DMs (top) and DEGs (left) in stage 2 vs. 3 of *A. truncatum* seed. The red indicates the positive correlation and green indicates the negative correlation. (**B**) KEGG pathway enrichment showed DMs and DEGs in stage 2 vs. 3 of *A. truncatum* seeds

The common enrichment pathway of DMs and DEGs was obtained by KEGG enrichment, which showed that DMs and DEGs were all enriched in ALA metabolism, biosynthesis of unsaturated fatty acids, glycerolipid metabolism, plant hormone signal transduction, zeatin biosynthesis and starch and sucrose metabolism in stage 1 vs. 2, stage 1 vs. 3, and stage 2 vs. 3 seeds (Fig. 3B & S6). The correlation between genes and metabolites was analyzed using cor function in R-analysis to calculate Pearson correlation coefficient. There were 71, 79 and 106 differential lipids in stage 1 vs. 2, stage 1 vs. 3 and stage 2 vs. 3, respectively (Fig. 3A & S7). And the number of differential lipids accounted for 21.26%, 17.91%, and 33.76% of all differentially expressed metabolites, respectively, which reflected important changes in lipids accumulation during *A. truncatum* seed development. Furthermore, the correlation coefficient greater than 0.80 and *p*-value less than 0.05 were selected for further analysis. ESA, ALA, and LA can be found with their correlated genes in stage 1 vs. 3 and stage 2 vs. 3, but just LA was correlated with genes between stage 1 and 2. The results suggested that DEGs were responsible for DMs, which is important for seed development and metabolites accumulation through the previously mentioned pathways in *A. truncatum*.

### The origin affected diversity of morphological characteristics and FAs content in *A. truncatum* seeds from 17 various locations

**Fig. 4:**
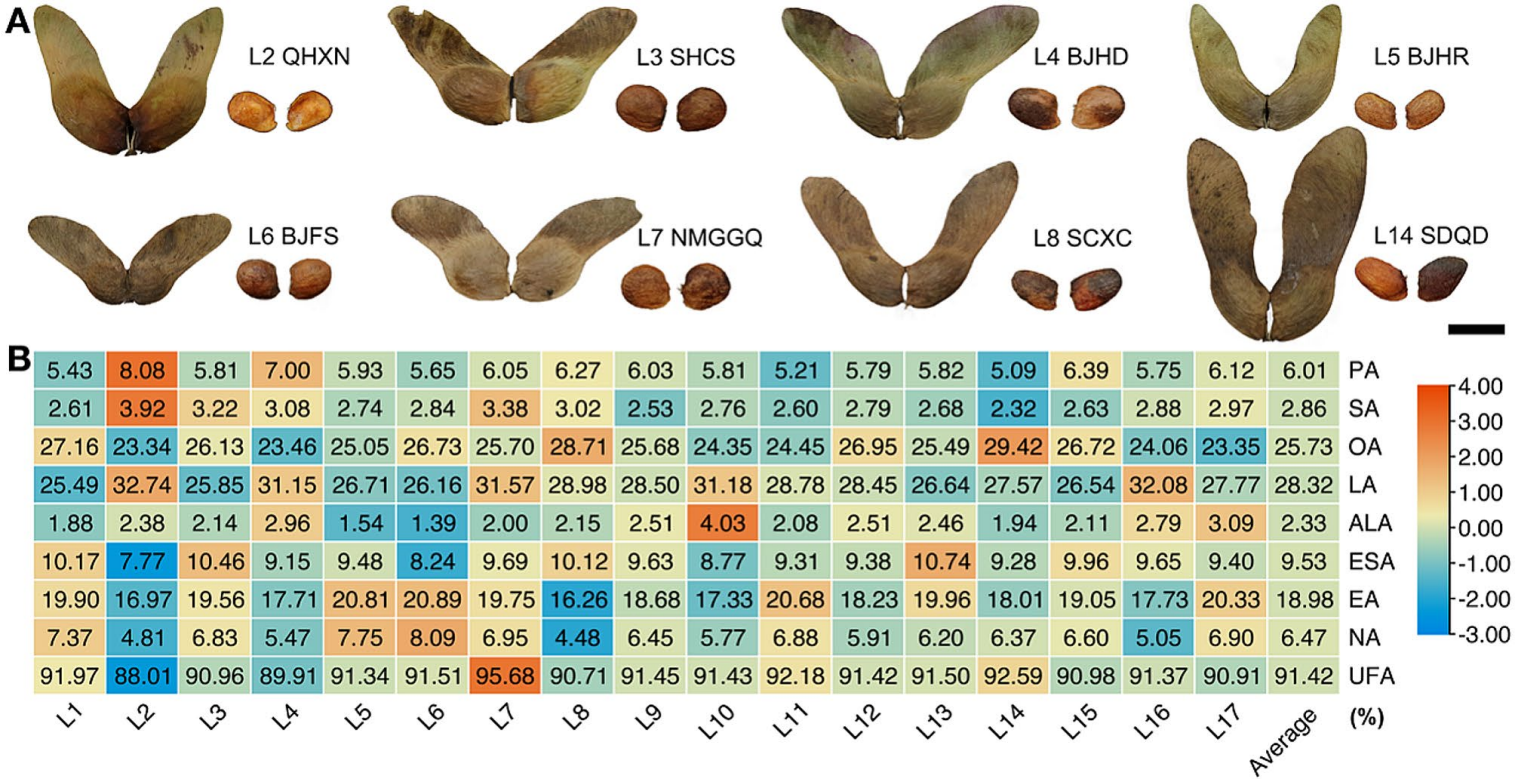
Morphological characteristics and eight main fatty acids of the *A. truncatum* stage 3 seeds (170 DAF) from sample locations. (**A**) Representative morphology of *A. truncatum* seeds sample from 8 sampling locations. (**B**) Relative content of 8 main FAs in *A. truncatum* seed from 17 different sampling locations. The scale bar refers to row scale

Inspecting transcriptional control on seed metabolites- especially FAs accumulation- showed that various factors, including environment, play an important role in seed traits such as phenotypes and the content of FAs. We have uncovered that the total FAs content was the highest at 170 DAF (stage 3), when the seeds are fully mature. Therefore, the seeds at stage 3 were collected to investigate the effect of environmental factors. In this study, different origins of seeds from 17 locations (L1–17, Table S5) demonstrated large diversity. Our data showed that the wing length and width of samara varied from 23.72 (L6) and 8.87 (L11), to 34.61 (L14) and 11.71 mm (L15), with an average of 27.79 and 10.02 mm, respectively. Moreover, the seed length, width, and thickness ranged from 7.39 (L5), 5.80 (L5), 2.43 (L5), to 10.36 (L14), 7.86 (L7) and 4.12 mm (L9), with an average of 9.19, 6.83 and 3.54 mm, respectively. Further, the 100-seed weight ranged from 4.87 (L4) to 14.80 g (L7), and the average weight was 10.01 g. The seed coat rate ranged from 28.41% (L9) to 49.20% (L5), and the average rate was 37.39%, indicating that the coat accounted for between 1/3 and 1/2 of the seed (Table S6, Fig. 4A).

Similarly, the content of FAs in the *A. truncatum* seeds from 17 sample locations also demonstrated a wide variation range: the average content of UFAs was up to 91.42%, and all FAs were significant differences (*p* < 0.05). Among these FAs, ALA and NA varied from 1.39% (L6) and 4.48% (L8) to 4.03% (L10) and 8.09% (L6), with an average of 2.33% and 6.47%, respectively. LA is the most abundant form of FAs. Its content ranged from 25.49% (L1) to 32.74% (L2), with an average of 28.32%. LA is followed by OA from 23.34% (L2) to 29.42% (L14), EA from 16.26% (L8) to 20.68% (L11), and ESA from 7.77% (L2) to 10.74% (L13), with an average of 25.73%, 18.98%, and 9.53%, respectively (Table S7, Fig. 4B).

### Bioclimatic factors showed significant influence on FAs content in *A. truncatum* seeds from 17 locations

**Fig. 5:**
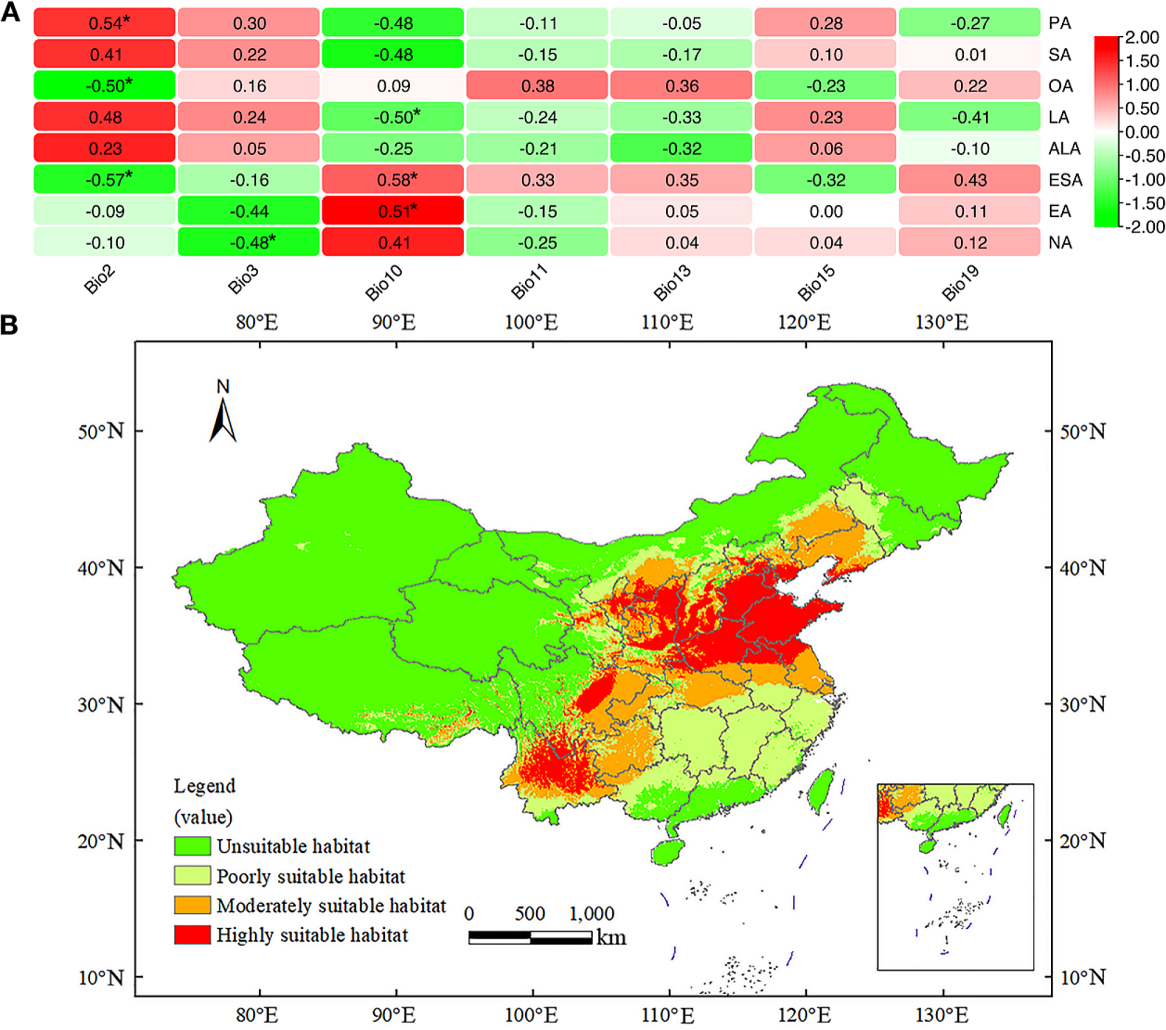
The correlation analysis between bioclimatic factors and relative contents of seed FAs and potential habitats of *A. truncatum*. (**A**) The relationship between bioclimatic factors and relative contents of *A. trunactum* seed FAs collected from different locations. The scale bar refers to row scale. (**B**) Modeling environmentally suitable areas for the potential habitats of *A. truncatum*. The figure is based on the prediction of the maximum entropy model using MaxEnt software for species habitat modeling (v3.4.4), and the map was generated by ArcMap 10.7 (ESRI, Redlands, CA, USA, http://www.esri.com/)

To further explore the effect of different bioclimatic factors on seed FAs content, we selected 19 bioclimatic variables for further study (Bio1–19, Table S8). After determination of bioclimatic variables using MaxEnt model, ArcGIS and SPSS analysis, 7 bioclimatic variables were selected to build the final MaxEnt model: Bio2, 3, 10, 11, 13, 15 and 19 (Table 1). According to the results of correlation analysis, the influence of each bioclimatic factor on the content of FAs in *A. truncatum* seeds varied greatly (Fig. 5A). The contents of OA and LA, which are the most abundant FAs in *A. truncatum* seeds, were significantly influenced by Bio2 (mean diurnal range, *r* = -0.50) and Bio10 (mean temperature of the warmest quarter, *r* = -0.50), respectively. In addition, the content of ESA was also negatively correlated with Bio2 (*r* = -0.57) and positively correlated with Bio10 (*r* = 0.58). The content of PA was also positively correlated with Bio2 (*r* = 0.54). Moreover, Bio3 (isothermality) was the only key influence factor on the content of NA (*r* = -0.48), and there were no bioclimatic factors significantly correlated with SA and ALA.

### Modeling environmentally suitable areas for the potential habitats of *A. truncatum*

Considering *A. truncatum*’s importance as a diet vegetable oil, screening the potential habitats for planting and industrial production is vital. Therefore, we selected 7 bioclimatic variables based on the above analysis, combining 116 distribution locations data to construct a model for potentially ecological suitability regions using MaxEnt model (Fig. 5B). The suitable habitat areas were then divided into four classes: (i) highly suitable habitats (0.4986–0.9081), (ii) moderately suitable habitats (0.2742–0.4986), (iii) poorly suitable habitats (0.0962–0.2742), (iv) unsuitable habitats (0–0.0962). According to the map, the potential distribution area of *A. truncatum* is mainly located in 18 provinces/regions in China (probability > 0.2742): Inner Mongolia, Liaoning, Hebei, Beijing, Tianjin, Shandong, Henan, Anhui, Jiangsu, Shanxi, Shaanxi, Ningxia, Gansu, Hubei, Sichuan, Chongqing, Guizhou and Yunnan. Among these, Shandong, southern Hebei, Beijing, Tianjin, southern Liaoning, southern Shanxi, northern Shaanxi, Ningxia, southern Inner Mongolia, northern Henan, northern Jiangsu, northern Anhui, northern Hubei, middle Sichuan and northern Yunnan were most suitable habitat areas for *A. truncatum*.

**Fig. 6:**
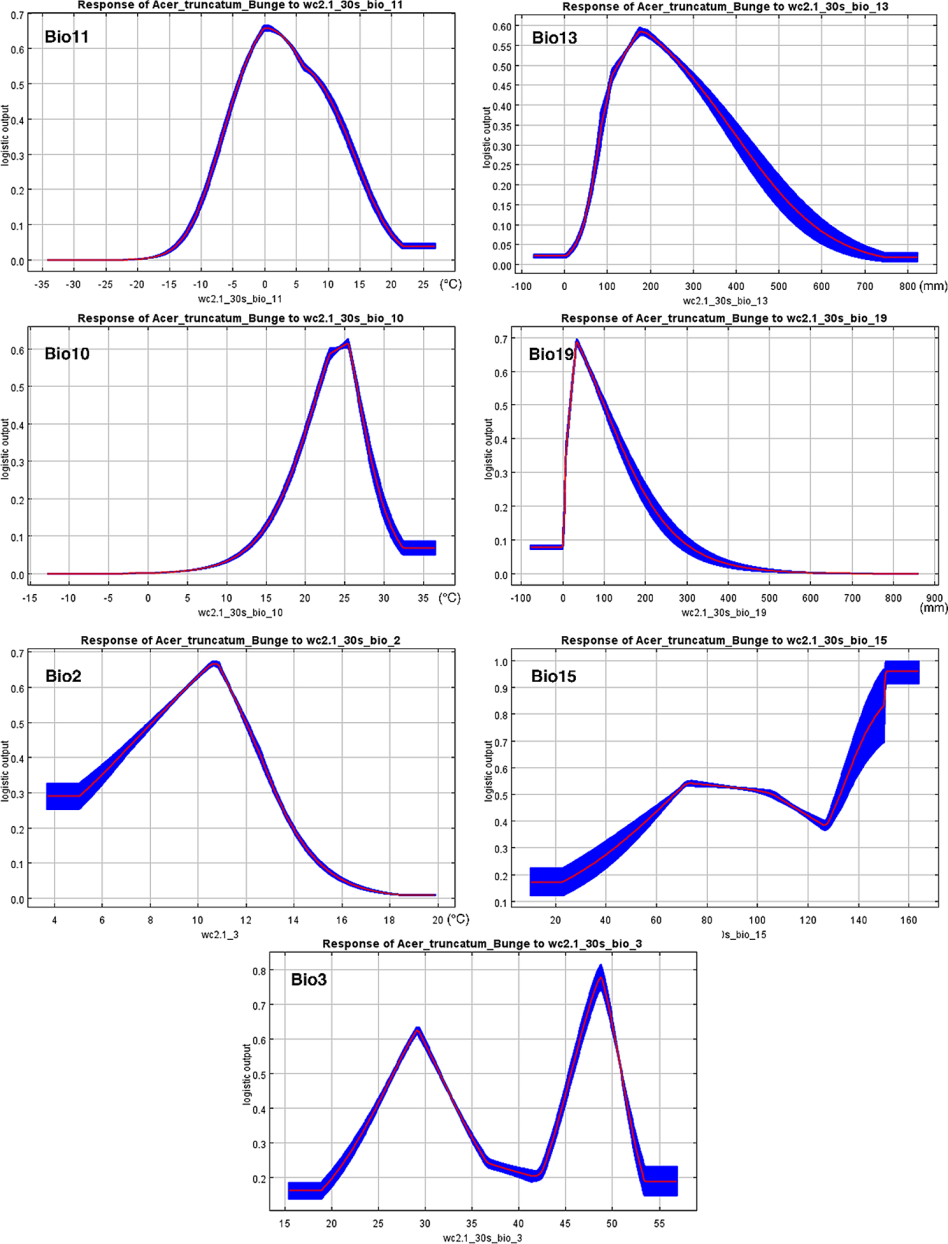
Response curves for selected 7 bioclimatic variables in the distribution model for *A. truncatum.* The curves show the mean response of the 10 replicate Maxent runs (red) and the mean ± SD (blue)

The accuracy of the simulation results is shown in Fig. S8. MaxEnt model predictions performed better than that of the random one [the area under ROC curve (AUC) = 0.5] and had high accuracy with a high mean training and testing AUC value, which was 0.902 and 0.879, respectively. The relative contribution of 7 bioclimatic variables is shown in Table 1. The four main bioclimatic variables affecting the potential distribution of *A. truncatum* are mean temperature of the coldest quarter (Bio11, 37.5%), precipitation of the wettest month (Bio13, 26.6%), mean temperature of the warmest quarter (Bio10, 18.1%) and precipitation of the coldest quarter (Bio19, 7.9%), with cumulative contribution as high as 90.1% (Fig. 6; Table 1).

## Discussion

Our study firstly monitored the accumulation pattern of FAs during seed development of *A. truncatum* and identified 95 (stage 1), 140 (stage 2) and 170 DAF (stage 3) as three key stages, which were the important time points for FAs accumulation. Moreover, after correlation analysis between transcriptome and metabolome in *A. truncatum* developmental seeds, we found FAs and their regulatory genes played a key role in seed development, especially *FAD3* (*Cluster-7222.41455*) expression level was increased from stage 1 to 2, but decreased at stage 3, which was consistent with the metabolic result of ALA. *KCS20* (*Cluster-7222.40643*) had the highest expression levels in *KCS*s family, and also highly expressed at stage 1–2, but decreased sharply at stage 3, which was consistent with the metabolic result of ESA, EA and NA. Furthermore, after evaluating regional differences of morphological characteristics and FAs content in *A. truncatum* seeds from 17 main production areas, we found that the seed from L5 was the smallest one among 17 locations’ seeds, and ALA and NA varied from 1.39% (L6) and 4.48% (L8) to 4.03% (L10) and 8.09% (L6). After analyzing the influences of 7 bioclimatic factors on FAs content of ATSO, we found Bio3 was the only key influence factors on the content of NA, but no bioclimatic factor had significant correlation with ALA. Using MaxEnt model based on 116 distribution locations data and 7 bioclimatic variables, we found the areas of 18 provinces were more suitable habitat for *A. truncatum* in China and Bio11, 13, 10 and 19 were the main four bioclimatic variables affecting its potential distribution .

Correlation analysis between transcriptome and metabolome has become a new effective way in the study of color, quality, growth and development, biotic or abiotic stress and other aspects. Recently, an integrative analysis of the transcriptome and metabolome was conducted in five *Camellia japonica* cultivars with different petal colors, and 187 flavonoids and 634 overlapping DEGs were identified, which provides new insights for elucidating the function of anthocyanins in *C. japonica* petal color demonstration [24]. ETHYLENE-INSENSITIVE 3/ETHYLENE-INSENSITIVE 3-LIKEs (EIN3/EILs) are important ethylene response factors during fruit ripening. Correlation analysis of transcriptome and metabolome data revealed that EIL2 is involved in β-carotene and ascorbic acid accumulation in tomato (*Solanum lycopersicum*), which could provide a future in strategy genetic engineering to improve the nutritional value and quality of tomato fruit [25]. Combined transcriptomic and metabolomic analysis between the staminate and pistillate flowers of mulberry revealed that DEGs and DMs may be associated with sex differentiation of mulberry through the regulation of the enrichment pathways, such as the MAPK pathway, which provides a rich source for the analysis of the molecular mechanism of mulberry sex differentiation processes [26]. In regards to stress tolerance, Guo et al. [27]. found the combination of drought and cold stress induced transcription-associated metabolomic alterations, in which, raffinose, trehalose-6-phosphate, and proline accumulated, and monosaccharide content was increased, and meanwhile, the increased abscisic acid (ABA) content and genes involved in ABA signaling pathway were upregulated, indicating the transcriptional regulation for the metabolic changes in maize. In this study, we carried out the correlation analysis between transcriptome and widely targeted metabolome in *A. truncatum* for the first time using developmental seed, the DMs and DEGs were all enriched in alpha-linolenic acid metabolism and biosynthesis of unsaturated fatty acids, etc. during seeds development, which provides a potential strategy for further study to improve the nutritional value and quality of ATSO. Furthermore, other methods of associative omics analysis have been developed, such as the integrated genome, transcriptome and metabolome analysis providing important resources for the research of kiwifruit functional genes, and molecular breeding [28]. Similar method was used to reveal the molecular mechanism of russeting in sand pear (*Pyrus pyrifolia* Nakai.) [29], which motivates us to pursue further research in plant development, metabolism, and stress with associative omics data.

ALA is the most abundant fatty acid in the chloroplast membrane of higher plants, which can capture photons in plant thylakoid membranes and plays an essential role in plant photosynthesis. In addition, ALA is also important in the plant’s ability to withstand adverse external factors such as cold temperatures, pests, and mechanical damage. It is catalyzed by ω-3 FADs, which are mainly divided into FAD3 located in ER and FAD7/FAD8 in plastid. It is widely believed that FAD3 is mainly responsible for the desaturation of stored oil in seeds and that it is also involved in the desaturation of membrane lipids in non-photosynthetic tissues (roots and flowers). FAD7 and FAD8 are mainly involved in the desaturation of chloroplast membrane lipids, which closely relate to plant tolerance of low and high temperatures [30]. In oil crops, the results are similar, since *FAD3A* is mainly responsible for the ALA accumulation in the seed, while *FAD7-1* and *FAD7-2* contribute mostly to the ALA present in the mesocarp by gene expression measurement and lipid analysis in *Olea europaea* [15]. In a new type of oil crop *Paeonia ostii*, *PoFAD3*, rather than *PoFAD7* or *PoFAD8*, plays a major role in the biosynthesis of ALA in seed [31]. In this study, we also found that *FAD3* overall had higher expression levels compared to that of *FAD7*/*8*, while, *AtFAD3* is closely related to *A. truncatum* seed ALA accumulation. However, we found 3 *FAD3* in seeds with different expression patterns, but needs further research in order to differentiate them. In plants, the expression of *KCS* determines whether VLCFAs are synthesized in specific cells. The expression of *KCS* genes, strict substrate specificity, and tissue specificity are directly and closely related to the biosynthesis of NA. KCS is the rate-limiting enzyme of the NA biosynthesis pathway [32, 33]. *KCS11* from *Malania oleifera* has a substrate preference for ESA, but not for EA to produce NA [34]. In *A. truncatum*, Ma et al. [17]. found that expression levels of most *KCS* genes were high during the early seed development and then gradually decreased. *Chr4.2307.KCS*, *Chr4.2308.KCS* and *Chr4.2311.KCS*, which all belong to *KCS2*/*20* subfamily, might be mainly responsible for regulating NA biosynthesis. In this study, we also found that *KCS20* (*Cluster-7222.40643*) had the highest expression levels, which was highly expressed at stage 1–2, but decreased sharply at stage 3. This is consistent with the VLCFA metabolism, including NA accumulation. However, the specific role of KCSs including KCS20 in the NA biosynthesis pathway in *A. truncatum* are still unclear and therefore needed to be further studied through enzyme activity detection and further experimentation.

Lipids in the seed of plant are crucial in controlling seed dormancy and growth, dispersal and defenses against biotic and abiotic stress [35]. Its biosynthesis and accumulation are affected by various natural environmental conditions. The kinds and contents of fatty acids are especially sensitive in colder climates [36]. In turn, the fatty acid composition may restrict the geographical distributions of plants to a significant extent [37]. Typically, higher proportions of unsaturated fatty acids help plants distribute in higher latitudes and altitudes, due to the promotion of germination earlier, and the growth being more rapid at lower temperatures [37]. Similar results were obtained in this study; we found that the content of UFAs including OA, ESA and NA had a significantly negative correlation with Bio2 or Bio3, which are related to temperature. The results provide very strong evidence to explain the content of the UFAs correlated with at least one pair of climatic condition groups. Since lipids or FAs of seeds are important for both plants and human needs, it is important to seek potential production areas for seed yields.

Species distribution models (SDMs) are ecological models that study the statistical relationship between species occurrence records and environmental factors and help determine the set of environmental conditions that are suitable for a species distribution [38, 39]. In recent years, SDMs have become an effective tool to predict the distribution patterns and potential habitat suitability assessments in many species [40–42]. In this study, the result indicated that under the current climate conditions, the potential distribution area of *A. truncatum* was located mainly in 18 provinces/regions in China. The *A. truncatum* in the high suitability areas are concentrated in the north and southwest of China, including Shandong, Hebei, Liaoning, Anhui, Sichuan, and Yunnan provinces, etc. These results of habitat suitability are consistent with the understanding of plant physiology, ecology and economics of *A. truncatum*, which is very important to guide the site selection for artificial introduction and cultivation practice. In addition, the predicted results show that four key bioclimatic variables including mean temperature of the coldest quarter (Bio11, 37.5%), precipitation of the wettest month (Bio13, 26.6%), mean temperature of the warmest quarter (Bio10, 18.1%) and precipitation of the coldest quarter (Bio19, 7.9%) affecting the potential distribution of *A. truncatum*, which indicated that temperature and precipitation was the most important factors. Our results indicated that when the mean temperature of the coldest quarter is lower than −20℃ or the mean temperature of the warmest quarter is lower than 5℃, the probability of the presence of *A. truncatum* is close to zero (Fig. 6), which reflects its cold tolerance and overwintering ability. The results of optimal precipitation of the wettest month (about 180 mm, Fig. 6), indicate that *A. truncatum* has strong drought tolerance. These findings provide scientific advice for *A. truncatum* managers to develop short-term and long-term adaptive strategies for forestry development planning in a rapidly changing climate.

## Materials and methods

### Plant materials and sampling location information

For FAs accumulation pattern analysis, the *A. truncatum* plants were grown in Beijing Botanical Garden, Institute of Botany, Chinese Academy of Sciences (Lat. 39°59’ N, 116°12’ E). Seeds were collected at six time points during their development: 95, 110, 125, 140, 155 and 170 DAF [12, 17]: on August 16th, August 31st, September 15th, September 30th, October 15th, and October 30th of 2021, respectively.

In order to investigate environmental effects on *A. truncatum* seeds, mature seeds (170 DAF) were collected from 17 locations in China from October to mid-November of 2021, and a GPS locator was used to record the latitude and longitude of each sampling location. Voucher specimens of *A. truncatum* seeds (2021:0185–0210) were deposited in the Herbarium, Institute of Botany, Chinese Academy of Sciences (Table S5). The collected seeds were kept at room temperature for seed morphology and FAs content analysis.

### Seed FAs component and content analysis

The method of seed FAs extraction and methyl-esterification followed the process previously described [31]. Then, the FAs content was determined by GC-MS (QP2010, Shimadzu, Japan). Eight FAs or fatty acid methyl ester (FAME) standards including PA, methyl stearate, methyl oleate, methyl linoleate, ALA, EA, NA, methyl *cis*-11-eicosenoate were purchased from Shanghai Yuanye Bio-Technology Co., Ltd (Shanghai, China). The retention time and area of each peak were used for seed FAs component and content analysis.

### Metabolite extraction and widely targeted metabolomic analysis

The metabolite extraction and widely targeted metabolomic analysis were performed based on the method previously reported [43]. The *A. truncatum* seeds at three key developmental stages were used to extract metabolites. The seed samples were freeze-dried by a vacuum freeze-dryer (Scientz-100 F) and then crushed using a mixer mill (MM 400, Retsch). The lyophilized powder sample of 100 mg was dissolved with 1.2 mL of 70% methanol/water (v/v) solution, then vortexed for 30 s every 30 min for 6 times, and finally incubated at 4°C overnight. The extracts were filtered by 0.22 μm pore size (SCAA-104, ANPEL, Shanghai, China) before Ultra-high performance liquid chromatography (UPLC)-MS/MS analysis (UPLC, SHIMADZU Nexera X2; MS, Applied Biosystems 4500 Q TRAP). The metabolites were identified through their mass-to-charge (m/z) values, retention times, and fragmentation patterns by Metware Biotechnology Co., Ltd. (Wuhan, China).

### DMs and KEGG pathway enrichment analysis

DMs between groups were selected by VIP ≥ 1 and absolute log_2_ fold change ≥ 1. After annotating metabolites by KEGG database, the pathways with significantly varied metabolites underwent enrichment analysis, and their significance was determined by hypergeometric test’s *p*-values.

### RNA extraction, cDNA library preparation and transcriptome assembly

The cDNA libraries were sequenced on the Illumina sequencing platform by Metware Biotechnology Co., Ltd. (Wuhan, China). Seeds of *A. truncatum* at three key developmental stages were used to extract RNA, and RNA integrity was evaluated using the RNA Nano 6000 Assay Kit of the Agilent Bioanalyzer 2100 system (Agilent Technologies, CA, USA). Sequencing libraries were generated using the NEBNext^®^ UltraTM RNA Library Prep Kit for Illumina^®^ (NEB, MA, USA), and the quality of the libraries was assessed using the Agilent Bioanalyzer 2100 system. The clustering of the index-coded samples was performed on a cBot Cluster Generation System using TruSeq PE Cluster Kit v3-cBot-HS (Illumina). To obtain clean data, reads with low quality or containing adapter and poly-N were filtered out. Then, Trinity (v2.11.0) was used for transcriptome assembly.

### RT-qPCR analysis

After extracting total RNA by E.Z.N.A.^®^ Plant RNA Kit (Omega Bio-Tek, GA, USA), the cDNA templates for RT-qPCR were made with HiScript II reverse transcriptase kit (Vazyme, Nanjing, China). Then, StepOne Real-Time PCR System (Applied Biosystems, Carlsbad, USA) and the 2× M5 HiPer Realtime PCR Super mix (Mei5bio, Beijing, China) were used for following reactions. *AtruActin* (*ACT*) was used as an internal control. The primers used in RT-qPCR are listed in Table S9.

### DEGs, gene ontology (GO), and KEGG pathway enrichment analysis

The DESeq2R package DESeq2 (1.10.1) was used for determining the DEGs. All DEGs were analyzed based on the GO term; on KEGG pathways, a hypergeometric distribution test was conducted with the pathway as the unit.

### Phylogenetic analyses

Phylogenetic analyses were performed as previously described [12, 17]. The phylogenetic trees of AtruFADs and AtFADs, and AtruKCSs and AtKCSs were constructed using the Maximum Likelihood method based on WAG + G and LG + G + I models, using MEGA software (vX) and Evolview (http://www.evolgenius.info/evolview.html), respectively. The analysis of AtruFADs and AtFADs, and AtruKCSs and AtKCSs was conducted with 20 and 41 amino acid sequences, respectively, and all positions with less than 80% site coverage were eliminated.

### Correlation analysis between genes expression levels and metabolites content

Quantitative values of genes and metabolites in all seed samples were used for correlation analysis. Using the cor function in R, we calculated the Pearson correlation coefficient of genes and metabolites. The results of correlation coefficient greater than 0.80 and *p*-value less than 0.05 were selected.

### Seed morphological characteristics analysis

Sixty samaras were randomly selected from each collection site. The wing length (the straight distance between the two wing tips) and wing width (the distance of parallel lines at the widest part of one wing) were measured with a vernier caliper (Ningbo Deli Tools Co., Ltd.). After the samara dried, the exocarp was removed, and the transverse diameter, longitudinal diameter and thickness of the seed were measured with vernier caliper to an accuracy of 0.01 mm. The 100-seed weight was investigated according to Chinese standards of GB/T 3543.7–1995. The seed coat rate was calculated by the following equation:

$$\eqalign{{\text{The}}\,{\text{seed}}\,{\text{coat}}\,{\text{rate}}(\% ) & = [{\text{the}}\,{\text{weight}}\,{\text{of}}\,{\text{the}}\,{\text{seed}}\,{\text{coat}}({\text{g}})] \cr & \quad /[{\text{the}}\,{\text{weight}}\,{\text{of}}\,{\text{the}}\,{\text{whole}}\,{\text{seed}}({\text{g}})] \times 100 \cr}$$

### Occurrence and distribution of *A. truncatum*

Three types of data were used to determine the occurrence and distribution of *A. truncatum*: (i) the 17 sampling locations previously mentioned; (ii) the online specimen information of *A. truncatum* in Chinese Digital Herbarium (https://www.cvh.ac.cn/); (iii) the distribution location data of *A. truncatum* provided by researcher Lin Zhang from Taishan Academy of Forestry Sciences. A total of 145 distribution locations were obtained, and the latitude and longitude details were obtained from Google Map (Table S10). The spatial analysis function of ArcGIS (v10.7) was used to determine the distance between the records and the center. This ensured that each censored grid only contained the distribution point closest to the center, therefore minimizing the effects of spatial autocorrelation. Ultimately, 116 distribution locations were selected (Table S10).

### Determination of bioclimatic variables and evaluation of potentially optimal habitat of *A. truncatum*

Nineteen bioclimatic variables (Bio1-Bio19) at spatial resolutions of 1 km were derived from the latest climate data provided by WorldClim (http://www.worldclim.org/) (Table S8). To further select the most important bioclimatic factors, the following three steps were taken, inspired by previous studies [44]. Firstly, all the 19 bioclimatic variables and 116 distribution locations were imported into the MaxEnt model (v3.4.4), which obtained the contribution rate of every bioclimatic variable (Table S8). Secondly, the attribute values of the bioclimatic variables corresponding to 116 distribution locations were extracted using ArcGIS, and the correlation between bioclimatic variables was calculated by SPSS (v26) using Pearson correlation analysis (Table S11). When *r* > 0.8, the contribution rates of two bioclimatic variables were compared, and variables with lower contribution rates were eliminated. After the above steps, 7 bioclimatic variables were retained to build the final MaxEnt model, which were Bio2, 3, 10, 11, 13, 15, and 19 (Table 1).

Based on the selected 116 distribution locations and 7 bioclimatic variables, MaxEnt model was used as the algorithm to calculate the percent contribution rate and construct the model for current habitat distribution of *A. truncatum* in China. To construct a prediction model, drawing on a previous study [44], the proportion of test data was set as ‘Random seed’, the output format was logistic, the maximum iterations was 500, and the importance of bioclimatic variables was measured by the ‘Jackknife test’. The model ran ten times, and the receiver operating characteristic curve by ROC analysis was used to confirm the accuracy of MaxEnt model’ s prediction.

### Statistical analysis

Statistical significance and clustering analysis were both conducted by GraphPad Prism (v7) and SPSS. PCA was performed by statistics function prcomp within R, with the data being scaled to unit variance before PCA. Pearson correlation analysis was used to assess the relationship between fatty acids and bioclimatic factors based on the seed FAs content and 7 selected bioclimatic variables using SPSS. The correlation analysis between expression levels of four genes from transcriptome and RT-qPCR data was conducted using SPSS with the default arguments and Pearson correlation coefficient to evaluate statistical significance (**, *P* < 0.01).

**Table 1:** Selected bioclimatic variables used to predict potential optimal habitat of *A. truncatum*

Bioclimatic variables	Description	Unit	Percent contribution (%)
Bio11	Mean temperature of the coldest quarter	°C	37.5
Bio13	Precipitation of the wettest month	mm	26.6
Bio10	Mean temperature of the warmest quarter	°C	18.1
Bio19	Precipitation of the coldest quarter	mm	7.9
Bio2	Mean diurnal range	°C	4.3
Bio15	Precipitation seasonality	/	3.4
Bio3	Isothermality	/	2.1

*Notice* The percent contribution refers to the contribution rate of every bioclimatic variable in the MaxEnt model

### Electronic supplementary material

Below is the link to the electronic supplementary material.

Supplementary Material 1

## Data Availability

The datasets generated and/or analyzed during the current study are available in the NCBI Sequence Read Archive (SRA) repository, BioProject’s metadata is available at https://www.ncbi.nlm.nih.gov/sra/PRJNA1008280.
