# Supplementary material for: Integrated analysis of metabolome, transcriptome, and bioclimatic factors of Acer truncatum seeds reveals key candidate genes related to unsaturated fatty acid biosynthesis, and potentially optimal production area
Source: BMC Plant Biol. 2024 Apr 16;24:284. doi: 10.1186/s12870-024-04936-6 (PMC11020666; doi:10.1186/s12870-024-04936-6)
Supplement: Supplementary file 1 — Supplementary Material 1 [file 12870_2024_4936_MOESM1_ESM.pdf]

Supporting Information

Figure S1. Heatmap of relative metabolites contents.

Figure S2. PCA of metabolome and transcriptome data.

Figure S3. KEGG enrichment analysis of DMs from metabolome at three developmental stages of *A. truncatum* seed.

Figure S4. KEGG enrichment analysis of DEGs from transcriptome at three developmental stages of *A. truncatum* seed.

Figure S5. Relative expression patterns of four genes of *A. truncatum* seed at three developmental stages detected by RNA-seq and RT-qPCR.

Figure S6. KEGG pathways jointly enriched from DMs and DEGs in stage 1 *vs* 2 and stage 1 *vs* 3 of *A. truncatum* seed.

Figure S7. Correlation heatmap of all the DMs and DEGs in stage 1 *vs* 2 and stage 1 *vs* 3 of *A. truncatum* seed.

Figure S8. Testing and training AUC values of ten-fold cross-validation models.

Table S1. The total number of differential metabolites and differentially expressed genes at three developmental stages of *A. truncatum* seed.

Table S2. The transcriptome basic data of *A. truncatum* seeds at three developmental stages.

Table S3. The expression levels of *FAD*s of *A. truncatum* seeds at three developmental stages.

Table S4. The expression levels of *KCS*s of *A. truncatum* seeds at three developmental stages.

Table S5. Location of the *A. truncatum* seed collected from 17 production areas.

Table S6. The seed morphological characteristics of the *A. truncatum* from 17 sampling locations.

Table S7. Relative content of eight main fatty acids in *A. truncatum* seed from 17 different sampling locations.

Table S8. Nineteen bioclimatic variables used in this study and their percent contribution.

Table S9. The primers used in RT-qPCR.

Table S10. The latitude and longitude details for recorded locations.

Table S11. Pairwise Pearson's correlation coefficients of bioclimatic variables.

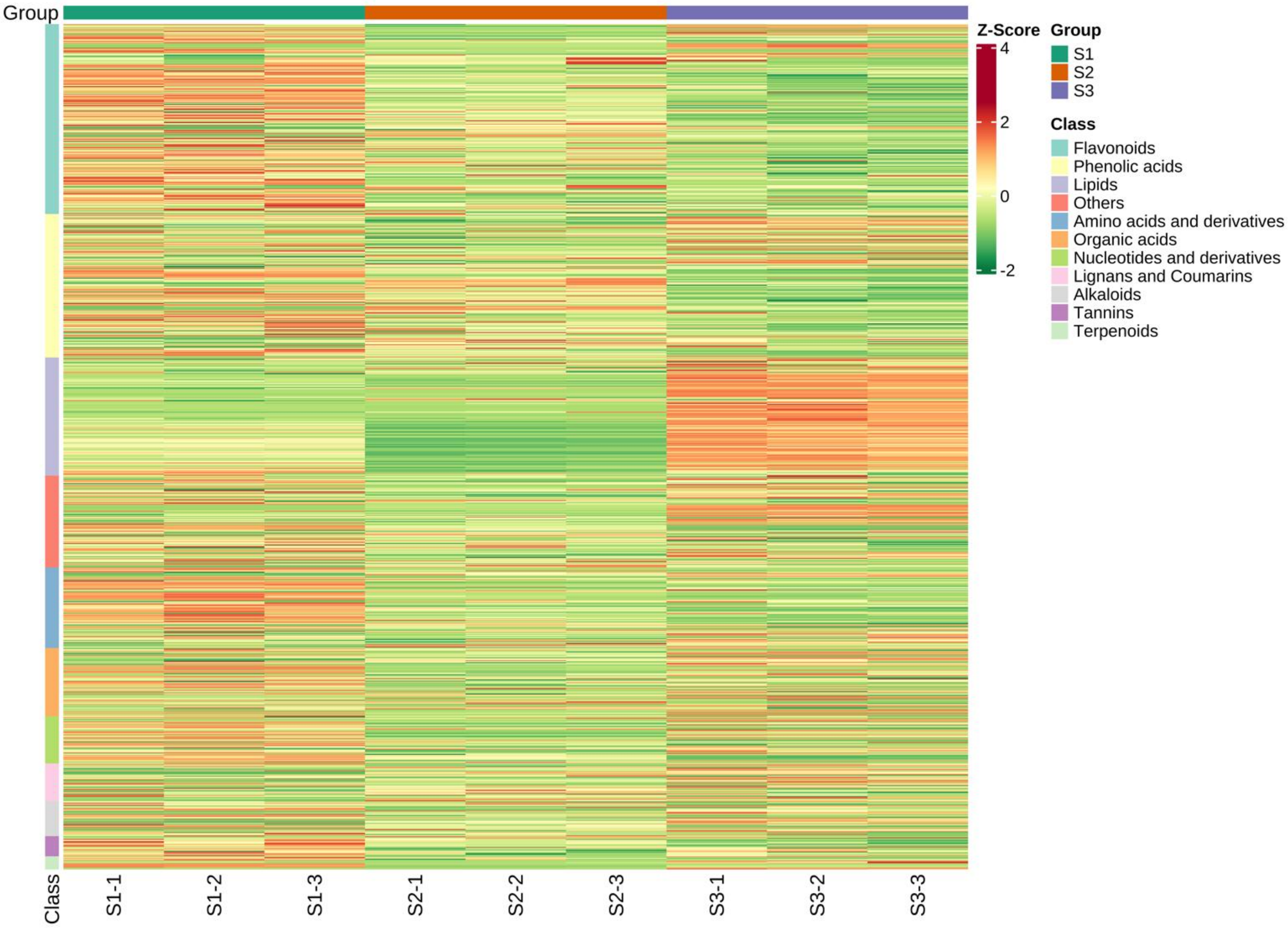

Figure S1. Heatmap of relative metabolites contents. X-axis indicates the sample name and the y-axis are the metabolites. Group indicates sample groups. The different colors are the results after standardization of the relative contents. S1, stage 1; S2, stage 2; S3, stage 3.

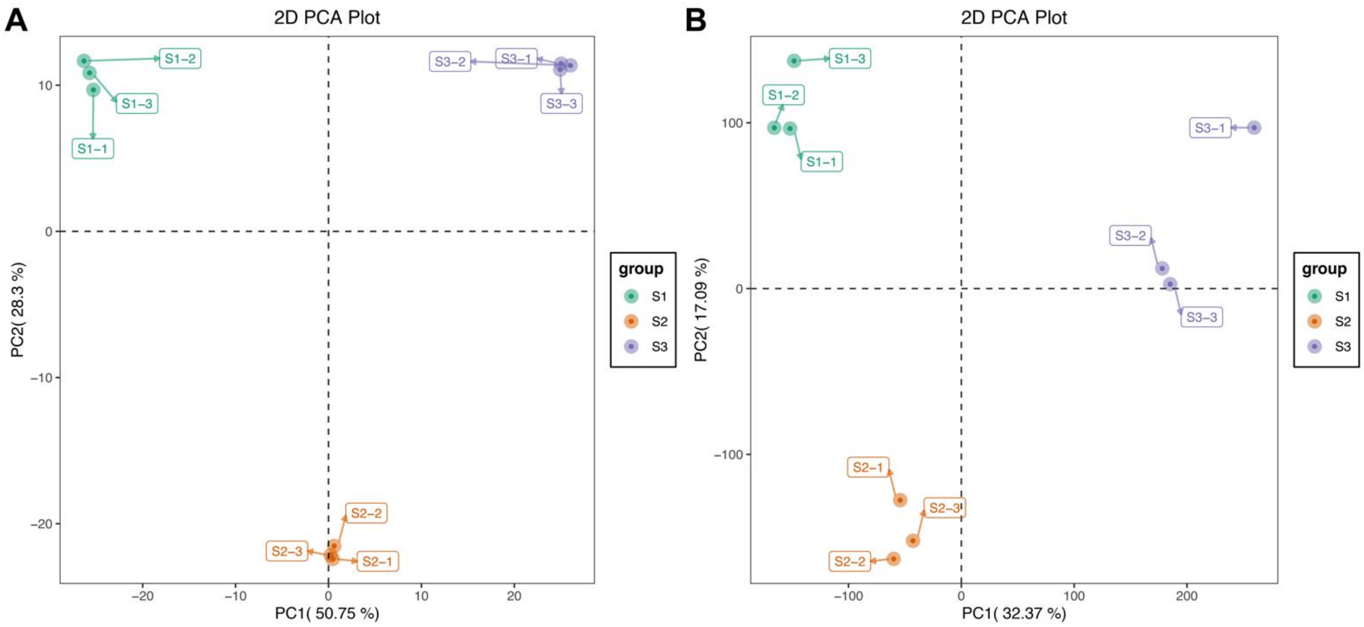

Figure S2. (A) PCA of metabolome and (B) transcriptome data. The x- and y-axis represents principal component 1 (PC1) and 2 (PC2), respectively. S1, stage 1; S2, stage 2; S3, stage 3.

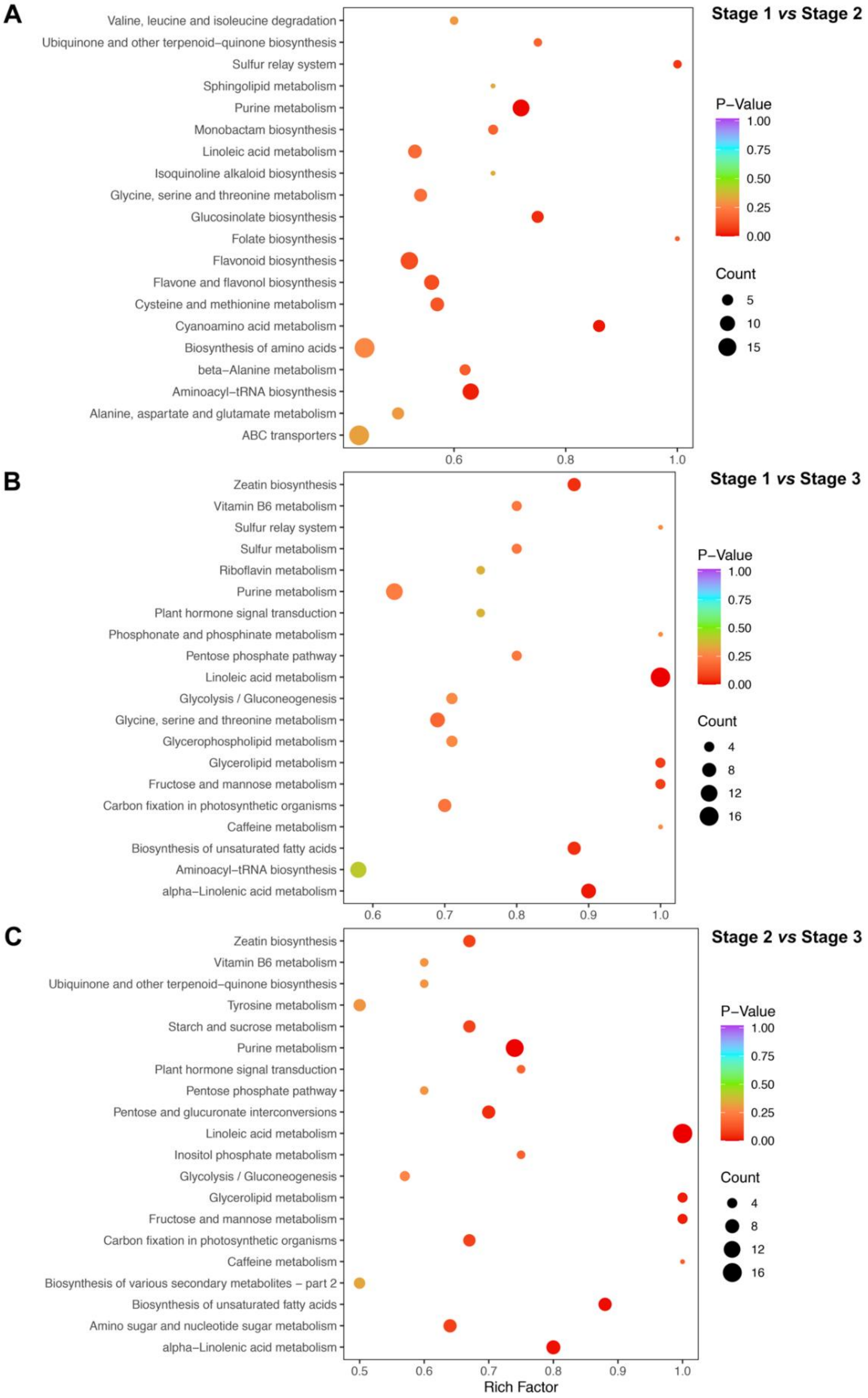

Figure S3. KEGG enrichment analysis of DMs from metabolome at three developmental stages of *A. truncatum* seed. KEGG pathway enrichment showed that DMs in (A) stage 1 *vs* 2, (B) stage 1 *vs* 3, and (C) stage 2 *vs* 3 seeds.

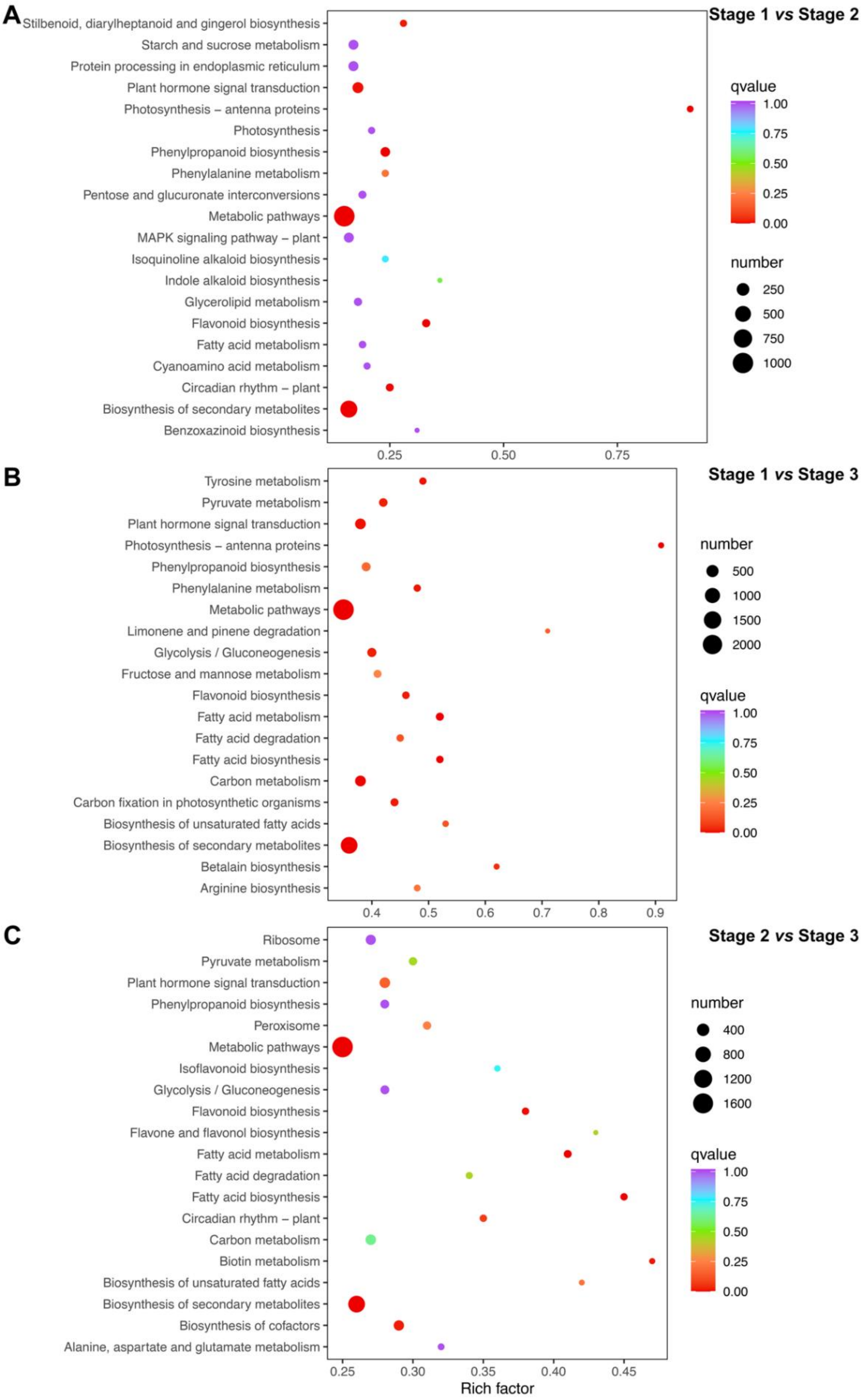

Figure S4. KEGG enrichment analysis of DEGs from transcriptome at three developmental stages of *A. truncatum* seed. KEGG pathway enrichment showed that DEGs in (A) stage 1 *vs* 2, (B) stage 1 *vs* 3, and (C) stage 2 *vs* 3 seeds.

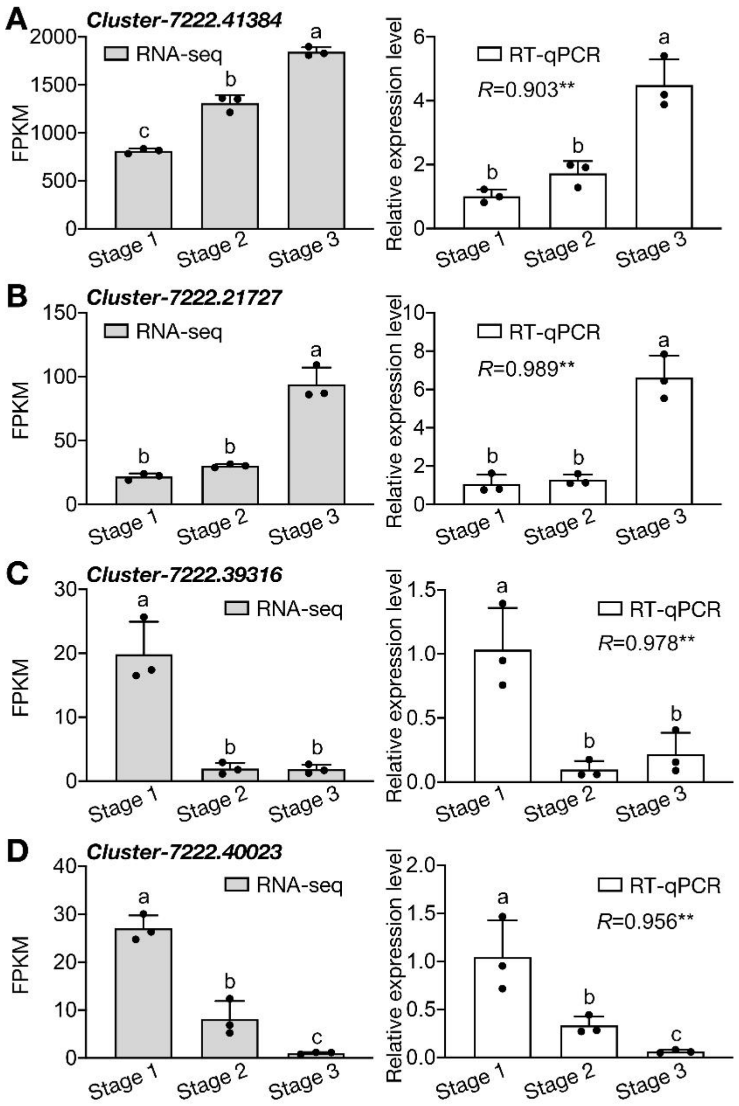

Figure S5. Relative expression patterns of four genes of *A. truncatum* seed at three developmental stages detected by RNA-seq (left) and RT-qPCR (right). Four genes were randomly selected from *FADs* and *KCSs*, which were increased (*Cluster-7222.41384* and *Cluster-7222.21727*) or decreased (*Cluster-7222.39316* and *Cluster-7222.40023*) its expression during seed development. The mean values ± SD are shown from three biological replicates (n = 3). Different letters above the columns represent statistically significant differences according to one-way ANOVA with Tukey's multiple comparisons test (P < 0.05). R refers to the correlation analysis between transcriptome data and RT-qPCR data was implemented with the default arguments and Pearson correlation coefficient to evaluate statistical significance (**, P < 0.01).

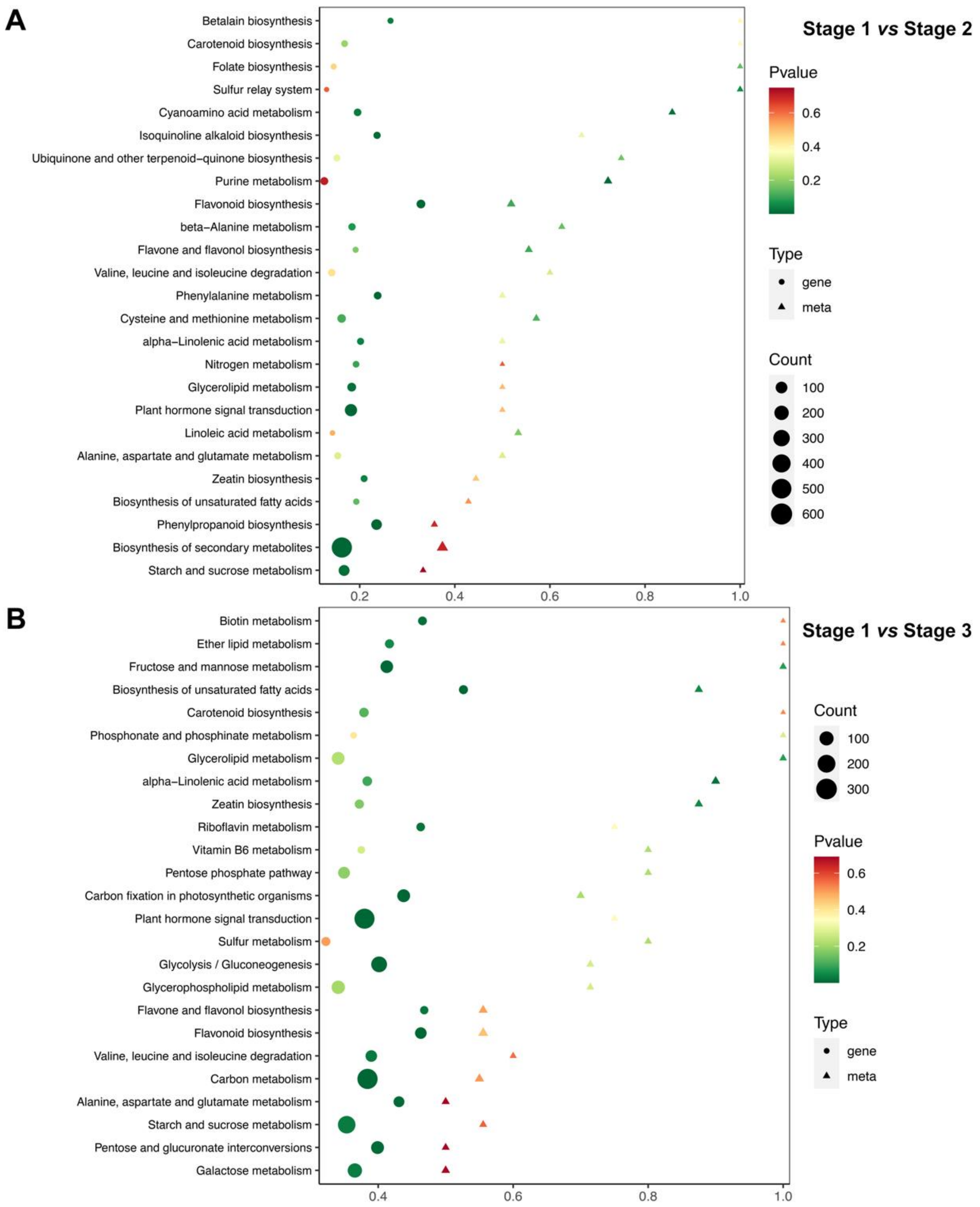

Figure S6. KEGG pathways jointly enriched from DMs and DEGs in (A) stage 1 *vs* 2 and (B) stage 1 *vs* 3 of *A. truncatum* seed.

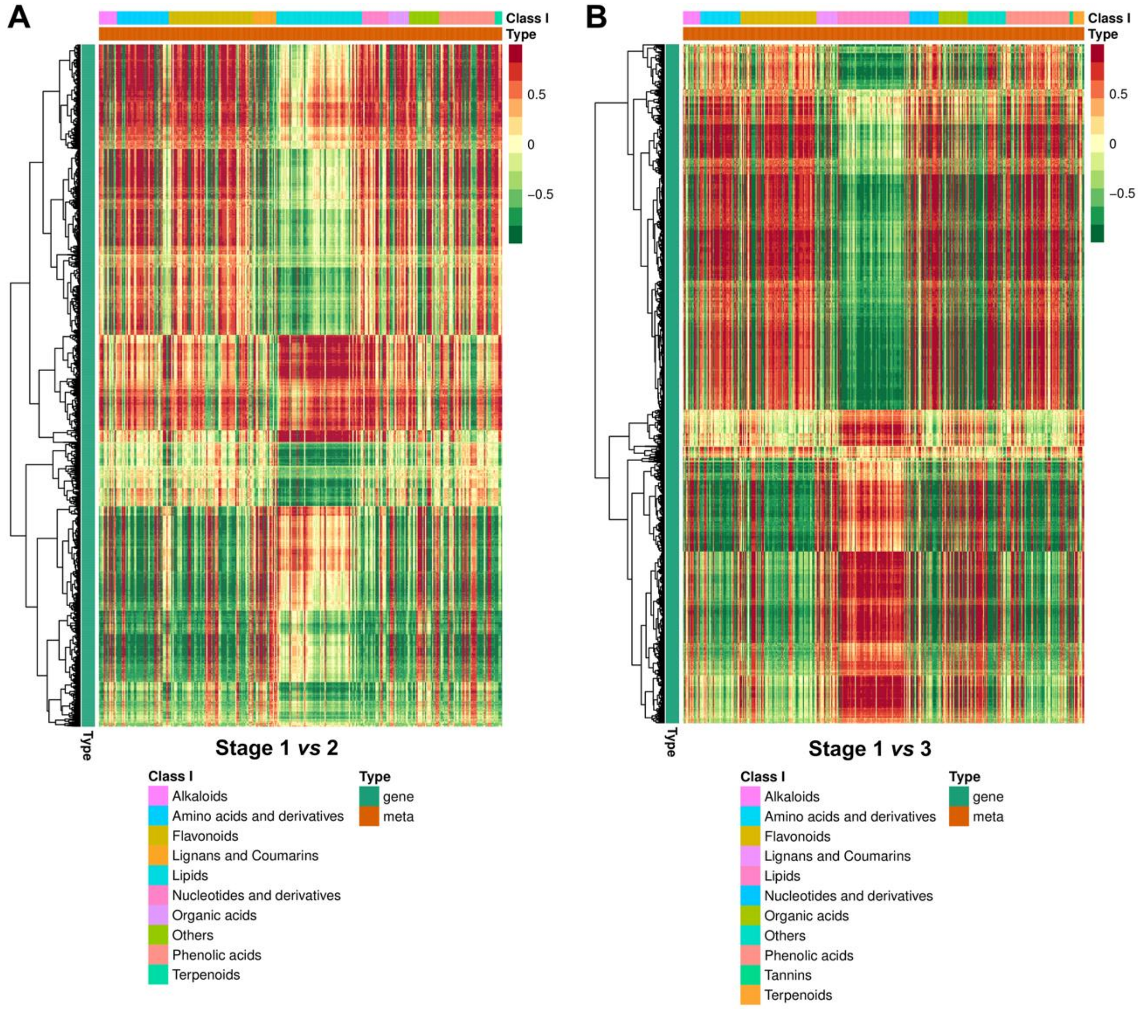

Figure S7. Correlation heatmap of all the DMs (top) and DEGs (left) in (A) stage 1 *vs* 2 and (B) stage 1 *vs* 3 of *A. truncatum* seed. the red indicates the positive correlation and green indicates the negative correlation.

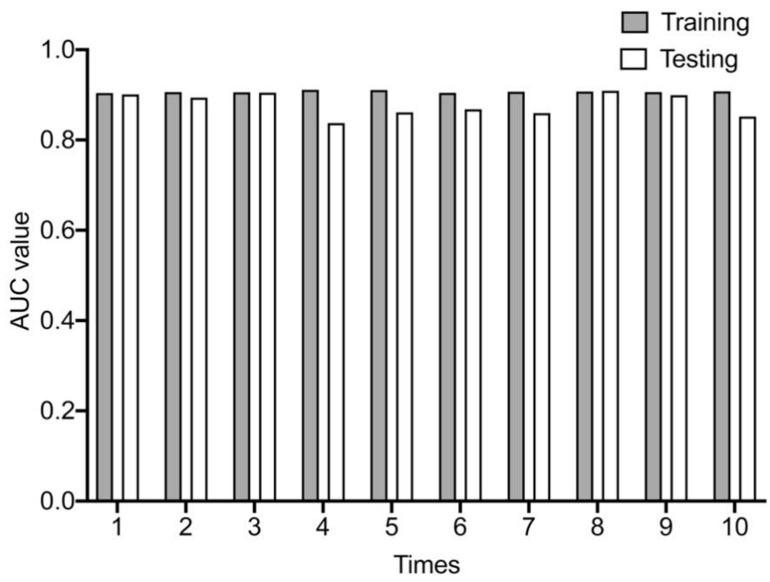

Figure S8. Testing and training AUC values of ten-fold cross-validation models. One to ten represented the model code, ascending in order by testing AUC value. The mean testing and training AUC values were 0.879 and 0.902, respectively.

Table S1. The total number of differential metabolites and differentially expressed genes at three developmental stages of *A. truncatum* seed.

Sample	DMs		DEGs	
	Up-regulated	Down-regulated	Up-regulated	Down-regulated
Stage 1 *vs* Stage 2	84	250	2868	3978
Stage 1 *vs* Stage 3	213	228	7428	8701
Stage 2 *vs* Stage 3	244	70	5997	5940

Table S2. The transcriptome basic data of *A. truncatum* seeds at three developmental stages.

Sample	Raw reads	Clean reads	Q20 (%)	Q30 (%)	GC (%)	Mapped reads	Mapped ratio (%)
Stage 1-1	48974158	47493590	98.08	94.51	46.41	40168106	84.58
Stage 1-2	47995100	46446798	98.03	94.35	46.32	39551880	85.16
Stage 1-3	48117228	46703494	98.08	94.51	46.19	40052368	85.76
Stage 2-1	43827378	42603822	98.3	95.02	47.11	36184236	84.93
Stage 2-2	47401646	45756156	98.23	94.79	46.38	39236380	85.75
Stage 2-3	47456918	45745978	98.21	94.76	46.84	38879436	84.99
Stage 3-1	44387160	42481824	97.91	94.26	45.54	34162072	80.42
Stage 3-2	43650466	41772448	98.04	94.5	46.23	33948058	81.27
Stage 3-3	49789452	47052074	98.07	94.56	45.94	38247112	81.29
Average	46844390	45117354	98.11	94.58	46.33	37825516	83.79

Table S3. The expression levels of *FADs* of *A. truncatum* seeds at three developmental stages.

Gene ID	Gene name	Annotation	FPKM (mean)		
			Stage 1	Stage 2	Stage 3
Cluster-7222.2877	*FAD4*	Fatty acid desaturase 4	0.31	1.31	0.80
Cluster-7222.41455	*FAD3*	ω3 fatty acid desaturase	72.32	94.32	33.54
Cluster-7222.15845	*FAD3*	ω3 fatty acid desaturase	8.04	4.63	14.23
Cluster-7222.57586	*FAD7*	ω3 fatty acid desaturase	1.17	0.55	0.07
Cluster-7222.37329	*FAD8*	ω3 fatty acid desaturase	2.30	0.88	0.21
Cluster-7222.53869	*FAD3*	ω3 fatty acid desaturase 3	0.44	0.64	0.00
Cluster-7222.41384	*FAD2*	ω6 fatty acid desaturase	812.61	1309.48	1845.99
Cluster-7222.27320	*FAD2*	ω6 fatty acid desaturase	590.29	637.60	87.25
Cluster-7222.33081	*FAD2*	ω6 fatty acid desaturase	287.00	249.77	50.14
Cluster-7222.21727	*FAD6*	ω6 fatty acid desaturase	21.94	30.31	94.07
Cluster-7222.48165	*FAD6*	ω6 fatty acid desaturase	1.38	0.81	2.45
Cluster-7222.23918	*FAD6*	ω6 fatty acid desaturase	0.85	0.19	1.60
Cluster-7222.26615	*FAD6*	ω6 fatty acid desaturase	0.00	0.38	0.11

Table S4. The expression levels of *KCSs* of *A. truncatum* seeds at three developmental stages.

Gene ID	Gene name	Annotation	FPKM (mean)		
			Stage 1	Stage 2	Stage 3
Cluster-12675.0	*KCS1*	Very-long-chain 3-ketoacyl-CoA synthase	0.28	0.86	0.11
Cluster-7222.27670	*KCS2*	3-ketoacyl-CoA synthase 2	1.08	3.85	0.65
Cluster-7222.39316	*KCS4*	3-ketoacyl-CoA synthase 4	19.87	1.95	1.87
Cluster-7222.39914	*KCS4*	Very-long-chain 3-ketoacyl-CoA synthase	4.28	2.40	4.00
Cluster-7222.9843	*KCS4*	3-ketoacyl-CoA synthase 4	6.07	2.09	4.30
Cluster-7222.26895	*KCS7*	3-ketoacyl-coa synthase 7	2.29	3.25	3.17
Cluster-7222.51503	*KCS7*	3-ketoacyl-coa synthase 7	1.04	1.27	5.59
Cluster-7222.40023	*KCS10*	3-ketoacyl-CoA synthase 10 -like protein	27.04	8.16	1.03
Cluster-7222.30172	*KCS11*	3-ketoacyl-CoA synthase 11	0.23	0.26	0.00
Cluster-7222.40366	*KCS11*	3-ketoacyl-CoA synthase 11	0.51	0.13	0.00
Cluster-7222.41581	*KCS11*	3-ketoacyl-CoA synthase 11	5.52	3.02	1.26
Cluster-7222.46486	*KCS11*	PREDICTED: 3-ketoacyl-CoA synthase 11-like	2.86	4.22	4.83
Cluster-8509.0	*KCS11*	3-ketoacyl-CoA synthase 11	0.95	0.30	0.38
Cluster-7222.27671	*KSC11*	PREDICTED: 3-ketoacyl-CoA synthase 11-like	0.23	0.57	0.25
Cluster-7222.18812	*KCS20*	3-ketoacyl-CoA synthase 20	0.71	3.03	0.61
Cluster-7222.37363	*KCS20*	3-ketoacyl-CoA synthase 20	27.96	26.06	27.75
Cluster-7222.40344	*KCS20*	3-ketoacyl-CoA synthase 20	1.47	0.38	0.00
Cluster-7222.40643	*KCS20*	3-ketoacyl-CoA synthase 20	1167.57	1705.46	81.46
Cluster-7222.40985	*KCS20*	3-ketoacyl-CoA synthase 20	12.18	11.64	18.06
Cluster-7222.43206	*KCS20*	3-ketoacyl-CoA synthase 20	4.64	7.23	51.80

Table S5. Location of the *A. truncatum* seed collected from 17 production areas.

Code	Location	Abbreviation	Latitude N (°)	Longitude E (°)	Information on the voucher specimen
L1	Shenyang, Liaoning province, China	LNSY	41.8051	123.4698	ATS LNSY 2021:0185
L2	Xining, Qinghai province, China	QHXN	36.6502	101.7662	ATS QHXN 2021:0186
L3	Songjiang district, Shanghai, China	SHCS	31.1211	121.385	ATS SHCS 2021:0187
L4	Haidian district, Beijing, China	BJHD	39.5923	116.124	ATS BJHD 2021:0188
L5	Huairou district, Beijing, China	BJHR	40.374	116.385	ATS BJHR 2021:0189
L6	Fangshan district, Beijing, China	BJFS	39.3917	115.535	ATS BJFS 2021:0190
L7	Tongliao, Inner Mongolia province, China	NMGGQ	43.6224	122.2561	ATS NMGGQ 2021:0191
L8	Xichang, Sichuan province, China	SCXC	27.8944	102.263	ATS SCXC 2021:0192
L9	Yuncheng, Shanxi province, China	SXYC	35.015	110.9981	ATS SXYC 2021:0193
L10	Fuxin, Liaoning province, China	LNFX	42.0133	111.6561	ATS LNFX 2021:0194
L11	Xianyang City, Shanxi province, China	SXXY	34.329	108.7064	ATS SXXY 2021:0195
L12	Kunming City, Yunnan province, China	YNKM	24.8855	102.8214	ATS YNKM 2021:0196
L13	Handan City, Hebei province, China	HBHD	36.6185	114.4933	ATS HBHD 2021:0197
L14	Qingdao City, Shandong province, China	SDQD	36.3074	120.3962	ATS SDQD 2021:0198
L15	Taian City, Shandong province, China	SDTA	36.1313	117.0906	ATS SDTA 2021:0199
L16	Liaocheng, Shandong province, China	SDLC	36.4345	115.9884	ATS SDLC 2021:0200
L17	Jining, Shandong province, China	SDJN	35.5811	116.5907	ATS SDJN 2021:0201

Table S6. The seed morphological characteristics of the *A. truncatum* from 17 sampling locations.

Code	Location	Wing		Seed			100-seed weight (g)	Seed coat rate (%)
		Length (mm)	Width (mm)	Length (mm)	Width (mm)	Thickness (mm)		
L1	LNSY	31.52±0.40^{b}	9.81±0.11^{def}	8.43±0.12^{gh}	6.24±0.07^{de}	3.65±0.15^{de}	6.43±0.39^{gh}	43.37±1.73^{bc}
L2	QHXN	28.85±0.25^{cde}	11.34±0.10^{ab}	9.99±0.11^{bc}	7.79±0.08^{ab}	3.49±0.11^{de}	5.59±0.24^{h}	46.84±0.33^{ab}
L3	SHCS	29.06±0.26^{cd}	10.37±0.11^{bcd}	10.28±0.11^{ab}	7.37±0.09^{abc}	3.40±0.09^{e}	9.05±0.29^{f}	39.90±0.44^{cd}
L4	BJHD	30.39±0.48^{bc}	10.79±0.18^{abc}	9.31±0.14^{ef}	7.05±0.09^{abcd}	3.47±0.11^{de}	4.87±0.63^{i}	39.18±0.99^{cd}
L5	BJHR	23.81±0.31^{i}	8.90±0.09^{def}	7.39±0.11^{i}	5.80±0.07^{e}	2.43±0.12^{g}	9.18±0.67^{f}	49.20±3.31^{a}
L6	BJFS	23.72±0.31^{i}	9.20±0.14^{ef}	8.20±0.10^{h}	6.37±0.07^{de}	3.62±0.14^{de}	6.38±0.48^{gh}	40.91±2.35^{cd}
L7	NMGGQ	28.32±0.41^{def}	11.27±0.19^{ab}	9.91±0.10^{cd}	7.86±0.08^{a}	4.07±0.07^{ab}	14.80±0.49^{a}	32.70±0.64^{fgh}
L8	SCXC	26.09±0.36^{gh}	9.31±0.10^{ef}	9.06±0.08^{f}	6.20±0.07^{de}	3.45±0.08^{de}	10.08±0.40^{ef}	37.13±1.17^{de}
L9	SXYC	25.35±0.37^{hi}	9.48±0.12^{def}	9.46±0.13^{e}	6.68±0.09^{cde}	4.12±0.08^{a}	13.56±0.15^{ab}	28.41±1.06^{i}
L10	LNFX	25.75±0.23^{gh}	9.75±0.14^{def}	9.08±0.13^{f}	6.85±0.08^{cd}	3.42±0.09^{de}	10.64±0.40^{de}	29.05±1.55^{hi}
L11	SXXY	25.05±0.18^{hi}	8.87±0.07^{f}	8.48±0.07^{gh}	6.59±0.05^{cde}	2.97±0.12^{f}	7.08±0.22^{g}	40.55±1.21^{cd}
L12	YNKM	27.21±0.30^{efg}	9.47±0.10^{def}	9.66±0.08^{cde}	6.32±0.06^{de}	3.77±0.13^{bcd}	11.63±0.31^{cd}	30.10±0.56^{ghi}
L13	HBHD	26.56±0.23^{gh}	9.22±0.07^{ef}	9.58±0.07^{de}	6.99±0.05^{bcd}	3.66±0.07^{de}	13.31±0.19^{b}	32.45±0.09^{ghi}
L14	SDQD	34.61±0.49^{a}	11.29±0.13^{ab}	10.36±0.11^{a}	6.69±0.06^{cde}	3.71±0.11^{cde}	11.73±0.12^{cd}	41.83±0.68^{c}
L15	SDTA	31.96±0.32^{b}	11.71±0.65^{a}	9.43±0.10^{e}	7.84±0.09^{a}	3.99±0.10^{abc}	13.63±0.29^{ab}	34.03±0.67^{efg}
L16	SDLC	27.14±0.34^{fg}	10.13±0.12^{cde}	9.04±0.19^{f}	6.83±0.06^{cd}	3.48±0.10^{de}	12.63±0.71^{bc}	36.83±0.77^{def}
L17	SDJN	27.07±0.42^{fg}	9.38±0.17^{ef}	8.61±0.13^{g}	6.61±0.11^{cde}	3.50±0.09^{de}	10.72±0.45^{de}	33.24±0.77^{efgh}
Range		23.72 - 34.61	8.87 - 11.71	7.39 - 10.36	5.80 - 7.86	2.43 - 4.12	4.87 - 14.80	28.41 - 49.20
Average		27.79±0.12	10.02±0.06	9.19±0.04	6.83±0.07	3.54±0.03	10.01±0.45	37.39±0.88

Notice: data were represented as mean of sixty different determinations ± SD. Different tiny letters in the same row indicate significant differences of $P < 0.05$.

Table S7. Relative content of eight main fatty acids in *A. truncatum* seed from 17 different sampling locations.

Code	Location	PA (%)	SA (%)	OA (%)	LA (%)	ALA (%)	ESA (%)	EA (%)	NA (%)	UFA (%)
L1	LNSY	5.43±0.04^{hi}	2.61±0.05^{hij}	27.16±0.04^{c}	25.49±0.83^{ef}	1.88±0.04^{def}	10.17±0.18^{abc}	19.90±0.40^{abcd}	7.37±0.23^{bcd}	91.97±0.09^{b}
L2	QHXN	8.08±0.12^{a}	3.92±0.14^{a}	23.34±0.29^{l}	32.74±1.26^{a}	2.38±0.16^{bcde}	7.77±0.16^{h}	16.97±0.67^{gh}	4.81±0.25^{ij}	88.01±0.15^{d}
L3	SHCS	5.81±0.13^{efg}	3.22±0.14^{bc}	26.13±0.15^{efg}	25.85±0.75^{cdef}	2.14±0.12^{bcdef}	10.46±0.17^{ab}	19.56±0.21^{abcdef}	6.83±0.21^{cdef}	90.96±0.20^{bc}
L4	BJHD	7.00±0.04^{b}	3.08±0.10^{cd}	23.46±0.25^{kl}	31.15±1.89^{abcd}	2.96±0.06^{bc}	9.15±0.29^{ef}	17.71±0.79^{efgh}	5.47±0.41^{ghij}	89.91±0.19^{c}
L5	BJHR	5.93±0.04^{efg}	2.74±0.04^{fghij}	25.05±0.16^{hi}	26.71±0.73^{ef}	1.54±0.14^{ef}	9.48±0.12^{cde}	20.81±0.41^{a}	7.75±0.31^{abc}	91.34±0.06^{bc}
L6	BJFS	5.65±0.08^{gh}	2.84±0.13^{defgh}	26.73±0.01^{cde}	26.16±1.46^{ef}	1.39±0.08^{f}	8.24±0.18^{gh}	20.89±0.68^{a}	8.09±0.50^{ab}	91.51±0.20^{bc}
L7	NMGGQ	6.05±0.17^{def}	3.38±0.09^{b}	25.70±0.52^{efg}	31.57±0.95^{abc}	2.00±0.12^{cdef}	9.69±0.21^{cde}	19.75±0.45^{abcd}	6.95±0.14^{cde}	95.68±2.23^{a}
L8	SCXC	6.27±0.07^{cd}	3.02±0.06^{cde}	28.71±0.17^{b}	28.98±0.93^{bcde}	2.15±0.08^{bcdef}	10.12±0.21^{abc}	16.26±0.56^{h}	4.48±0.32^{j}	90.71±0.12^{bc}
L9	SXYC	6.03±0.1^{def}	2.53±0.01^{ijk}	25.68±0.07^{fg}	28.50±0.07^{cde}	2.51±0.22^{bcde}	9.63±0.03^{cde}	18.68±0.14^{bcdefg}	6.45±0.10^{defg}	91.45±0.10^{bc}
L10	LNFX	5.81±0.13^{efg}	2.76±0.03^{fghi}	24.35±0.12^{j}	31.18±1.08^{abcd}	4.03±1.07^{a}	8.77±0.33^{fg}	17.33±0.97^{fgh}	5.77±0.45^{fghi}	91.43±0.10^{bc}
L11	SXXY	5.21±0.05^{ij}	2.60±0.07^{hij}	24.45±0.10^{ij}	28.78±0.55^{bcde}	2.08±0.03^{cdef}	9.31±0.04^{def}	20.68±0.14^{a}	6.88±0.25^{cde}	92.18±0.10^{b}
L12	YNKM	5.79±0.07^{efg}	2.79±0.10^{efgh}	26.95±0.09^{cd}	28.45±1.16^{cde}	2.51±0.15^{bcde}	9.38±0.17^{def}	18.23±0.68^{cdefg}	5.91±0.33^{efgh}	91.42±0.01^{bc}
L13	HBHD	5.82±0.11^{efg}	2.68±0.10^{ghij}	25.49±0.06^{gh}	26.64±1.36^{ef}	2.46±0.09^{bcde}	10.74±0.28^{a}	19.96±0.62^{abc}	6.20±0.37^{efg}	91.50±0.21^{bc}
L14	SDQD	5.09±0.09^{j}	2.32±0.02^{k}	29.42±0.10^{a}	27.57±0.44^{ef}	1.94±0.21^{def}	9.28±0.08^{def}	18.01±0.44^{defgh}	6.37±0.20^{defg}	92.59±0.10^{b}
L15	SDTA	6.39±0.14^{c}	2.63±0.08^{ghij}	26.72±0.09^{cde}	26.54±1.60^{ef}	2.11±0.13^{cdef}	9.96±0.32^{bcd}	19.05±0.90^{abcdef}	6.60±0.30^{def}	90.98±0.22^{bc}
L16	SDLC	5.75±0.03^{fg}	2.88±0.05^{defg}	24.06±0.16^{jk}	32.08±0.38^{ab}	2.79±0.12^{bcd}	9.65±0.05^{cde}	17.73±0.27^{efgh}	5.05±0.06^{hij}	91.37±0.06^{bc}
L17	SDJN	6.12±0.03^{cde}	2.97±0.03^{def}	23.35±0.28^{l}	27.77±0.76^{def}	3.09±0.27^{b}	9.40±0.29^{def}	20.33±0.49^{ab}	6.90±0.26^{cde}	90.91±0.06^{bc}
Range		5.09 - 8.08	2.32 - 3.92	23.34 - 29.42	25.49 - 32.74	1.39 - 4.03	7.77 - 10.74	16.26 - 20.68	4.48 - 8.09	88.01 - 95.67
Average		6.01±0.09	2.86±0.05	25.73±0.23	28.32±0.39	2.33±0.10	9.53±0.11	18.98±0.22	6.47±0.16	91.42±0.22

Notice: data were represented as mean of three different determinations ± SD. Different tiny letters in the same row indicate significant differences of $P < 0.05$.

Table S8. Nineteen bioclimatic variables used in this study and their percent contribution.

Bioclimatic variables	Description	Unit	Percent contribution (%)
Bio1	Annual mean temperature	°C	7.6
Bio2	Mean diurnal range [mean of monthly (max temperature - min temperature)]	°C	4.1
Bio3	Isothermality [(Bio2/Bio7) × 100]	/	1.9
Bio4	Temperature seasonality (standard deviation × 100)	°C	1.5
Bio5	Max temperature of the warmest month	°C	2.7
Bio6	Min temperature of the coldest month	°C	1.8
Bio7	Temperature annual range (Bio5 - Bio6)	°C	0.8
Bio8	Mean temperature of the wettest quarter	°C	1
Bio9	Mean temperature of the driest quarter	°C	2.9
Bio10	Mean temperature of the warmest quarter	°C	12.1
Bio11	Mean temperature of the coldest quarter	°C	22.6
Bio12	Annual precipitation	mm	1.7
Bio13	Precipitation of the wettest month	mm	24.1
Bio14	Precipitation of the driest month	mm	1.5
Bio15	Precipitation seasonality (coefficient of variation)	/	1.9
Bio16	Precipitation of the wettest quarter	mm	0.1
Bio17	Precipitation of the driest quarter	mm	3.1
Bio18	Precipitation of the warmest quarter	mm	3.6
Bio19	Precipitation of the coldest quarter	mm	4.9

Table S9. The primers used in RT-qPCR.

Gene ID	Forward primer (5'→3')	Reverse primer (5'→3')
AtruACT	GACAACCTCAAACAACGCGAAAT	AGCAAACCCAGCCTTAACCAT
Cluster-7222.41384	TGAGCCAGACGAAGGTAACCAGA	ATAAACAATTTAACAGCCTAAAGTACCAGG
Cluster-7222.21727	AGAATTGGGGAAAGTATATGAACGAGG	GAAATGGCAACTAATCTCAGGTGGG
Cluster-7222.39316	AGAAGGTGAAAAAGGGAGACAGAGT	CGTGATCGACAAAAGCACAAATATATG
Cluster-7222.40023	CTAGTGAAAAAACCCAGTTCAGGAATC	CCAAAGACGAGAAAAGTAGAGCAAA

Table S10. The latitude and longitude details for recorded locations.

No.	Species	Latitude N (°)	Longitude E (°)
1	*A. truncatum*	23.7056	102.4962
2	*A. truncatum*	24.0419	104.1958
3	*A. truncatum*	24.0700	101.9901
4	*A. truncatum*	24.4106	103.4150
5	*A. truncatum*	24.5033	117.7098
6	*A. truncatum*	24.6738	102.9082
7	*A. truncatum*	24.8855	102.8215
8	*A. truncatum*	25.5042	101.2416
9	*A. truncatum*	25.8231	98.8580
10	*A. truncatum*	26.1111	99.9509
11	*A. truncatum*	26.4057	106.2553
12	*A. truncatum*	26.4535	99.4167
13	*A. truncatum*	26.5601	100.1764
14	*A. truncatum*	26.6843	100.7509
15	*A. truncatum*	26.8772	100.2255
16	*A. truncatum*	27.1866	103.5579
17	*A. truncatum*	27.3203	103.7059
18	*A. truncatum*	27.4415	104.8736
19	*A. truncatum*	27.8469	105.0491
20	*A. truncatum*	27.8945	102.2631
21	*A. truncatum*	28.1086	104.2347
22	*A. truncatum*	28.2291	103.6382
23	*A. truncatum*	28.5984	103.9584

24	*A. truncatum*	28.9465	117.5788
25	*A. truncatum*	29.2697	88.8870
26	*A. truncatum*	29.4167	105.6119
27	*A. truncatum*	29.7055	116.0014
28	*A. truncatum*	29.9957	104.1341
29	*A. truncatum*	30.0422	103.8324
30	*A. truncatum*	30.1441	102.9283
31	*A. truncatum*	30.2084	120.2120
32	*A. truncatum*	30.5745	103.9238
33	*A. truncatum*	30.6598	104.1019
34	*A. truncatum*	30.9884	103.6466
35	*A. truncatum*	31.1211	121.3850
**36**	***A. truncatum***	**31.3160**	**112.5811**
37	*A. truncatum*	31.3284	120.8008
**38**	***A. truncatum***	**31.4350**	**121.1058**
39	*A. truncatum*	31.4769	103.5903
40	*A. truncatum*	31.8538	113.3000
41	*A. truncatum*	32.1319	119.4340
**42**	***A. truncatum***	**32.1512**	**119.8210**
**43**	***A. truncatum***	**33.1638**	**103.5524**
44	*A. truncatum*	33.2011	120.5010
**45**	***A. truncatum***	**33.2828**	**112.2551**
46	*A. truncatum*	33.2943	118.8735
**47**	***A. truncatum***	**33.3300**	**110.5110**
48	*A. truncatum*	33.4234	109.1526

**49**	***A. truncatum***	**33.4601**	**106.0441**
50	*A. truncatum*	33.4898	112.4291
**51**	***A. truncatum***	**33.5833**	**106.3912**
52	*A. truncatum*	33.7750	120.2582
53	*A. truncatum*	33.9627	118.2424
54	*A. truncatum*	34.1260	113.8227
55	*A. truncatum*	34.1513	109.3235
**56**	***A. truncatum***	**34.2007**	**107.5811**
57	*A. truncatum*	34.2305	108.9343
58	*A. truncatum*	34.2722	108.0846
**59**	***A. truncatum***	**34.3222**	**110.0532**
**60**	***A. truncatum***	**34.3231**	**110.0525**
61	*A. truncatum*	34.3291	108.7064
62	*A. truncatum*	34.3693	118.3555
**63**	***A. truncatum***	**34.4312**	**108.0348**
**64**	***A. truncatum***	**34.5032**	**110.2297**
65	*A. truncatum*	34.5276	108.2394
66	*A. truncatum*	34.5660	110.0921
67	*A. truncatum*	34.5702	105.8897
68	*A. truncatum*	34.6469	115.1477
69	*A. truncatum*	34.7714	111.1875
70	*A. truncatum*	34.8347	111.1947
71	*A. truncatum*	35.0151	110.9982
72	*A. truncatum*	35.0671	112.6027
73	*A. truncatum*	35.0691	109.0759

74	*A. truncatum*	35.0710	115.5730
75	*A. truncatum*	35.1134	117.1660
76	*A. truncatum*	35.4052	117.0039
77	*A. truncatum*	35.4862	112.4149
78	*A. truncatum*	35.5015	112.8535
79	*A. truncatum*	35.5808	116.9865
80	*A. truncatum*	35.5811	116.5907
81	*A. truncatum*	35.6647	117.2522
82	*A. truncatum*	35.7751	113.2806
**83**	***A. truncatum***	**35.9310**	**112.7410**
84	*A. truncatum*	36.1040	103.7188
**85**	***A. truncatum***	**36.1242**	**117.7160**
86	*A. truncatum*	36.1314	117.0906
87	*A. truncatum*	36.1920	117.1353
**88**	***A. truncatum***	**36.1958**	**113.1165**
89	*A. truncatum*	36.2001	113.4361
90	*A. truncatum*	36.3074	120.3963
91	*A. truncatum*	36.3155	112.8922
**92**	***A. truncatum***	**36.4415**	**111.4833**
93	*A. truncatum*	36.4947	117.8617
94	*A. truncatum*	36.5023	113.3872
95	*A. truncatum*	36.5345	104.1724
96	*A. truncatum*	36.5536	116.7520
97	*A. truncatum*	36.6185	114.4934
98	*A. truncatum*	36.6502	101.7663

99	*A. truncatum*	36.6546	119.1661
100	*A. truncatum*	36.7595	110.6320
101	*A. truncatum*	36.7765	121.1585
102	*A. truncatum*	36.8479	111.7788
103	*A. truncatum*	36.8637	109.3290
104	*A. truncatum*	36.8781	110.1938
105	*A. truncatum*	37.0271	111.9167
**106**	***A. truncatum***	**37.4642**	**113.2560**
107	*A. truncatum*	37.5176	111.1508
108	*A. truncatum*	37.5945	107.6013
109	*A. truncatum*	37.9404	112.4870
110	*A. truncatum*	38.4043	112.7462
111	*A. truncatum*	38.7305	112.7111
112	*A. truncatum*	38.9297	100.4780
113	*A. truncatum*	39.1890	113.2655
114	*A. truncatum*	39.3917	115.5350
115	*A. truncatum*	39.4704	75.9898
**116**	***A. truncatum***	**39.4900**	**119.3040**
117	*A. truncatum*	39.5124	112.2883
**118**	***A. truncatum***	**39.5800**	**116.1100**
**119**	***A. truncatum***	**39.5900**	**115.2500**
120	*A. truncatum*	39.5923	116.1240
121	*A. truncatum*	39.8351	119.4846
122	*A. truncatum*	40.0458	117.4083
**123**	***A. truncatum***	**40.2700**	**116.0000**

**124**	***A. truncatum***	**40.3300**	**117.2800**
125	*A. truncatum*	40.3663	113.7541
126	*A. truncatum*	40.3740	116.3850
127	*A. truncatum*	40.4568	115.9750
**128**	***A. truncatum***	**40.5035**	**118.4601**
129	*A. truncatum*	40.7753	120.8691
130	*A. truncatum*	40.8077	111.6230
131	*A. truncatum*	40.9748	115.2823
132	*A. truncatum*	40.9906	113.1240
133	*A. truncatum*	41.8052	123.4699
134	*A. truncatum*	42.0133	121.6562
135	*A. truncatum*	42.0133	111.6562
**136**	***A. truncatum***	**42.1733**	**118.5907**
**137**	***A. truncatum***	**42.1933**	**122.5046**
138	*A. truncatum*	42.3865	122.5389
139	*A. truncatum*	42.5468	124.1592
**140**	***A. truncatum***	**43.5336**	**125.1915**
141	*A. truncatum*	43.6224	122.2562
**142**	***A. truncatum***	**45.0242**	**121.2733**
**143**	***A. truncatum***	**45.1337**	**121.3012**
144	*A. truncatum*	45.5477	126.9572
145	*A. truncatum*	36.4345	115.9885

Notice: the locations marked by bold fonts were removed by ArcGIS analysis.

Table S11. Pairwise Pearson's correlation coefficients of bioclimatic variables.

	Bio1	Bio2	Bio3	Bio4	Bio5	Bio6	Bio7	Bio8	Bio9	Bio10	Bio11	Bio12	Bio13	Bio14	Bio15	Bio16	Bio17	Bio18	Bio19
Bio1	1																		
Bio2	-0.681	1																	
Bio3	0.283	0.258	1																
Bio4	-0.655	0.303	**-0.818**	1															
Bio5	0.430	-0.419	-0.618	0.383	1														
Bio6	**0.952**	-0.675	0.450	**-0.834**	0.167	1													
Bio7	-0.786	0.514	-0.676	**0.970**	0.204	**-0.931**	1												
Bio8	0.592	-0.589	-0.506	0.206	**0.923**	0.346	-0.003	1											
Bio9	**0.931**	-0.612	0.523	**-0.875**	0.095	**0.992**	**-0.950**	0.281	1										
Bio10	0.611	-0.577	-0.500	0.196	**0.966**	0.366	-0.006	**0.977**	0.294	1									
Bio11	**0.930**	-0.573	0.563	**-0.887**	0.079	**0.990**	**-0.953**	0.261	**0.996**	0.279	1								
Bio12	**0.840**	-0.725	0.339	-0.695	0.125	**0.864**	**-0.811**	0.398	**0.870**	0.353	**0.851**	1							
Bio13	0.602	-0.262	0.210	-0.271	0.287	0.466	-0.358	0.560	0.468	0.456	0.485	0.623	1						
Bio14	0.507	**-0.806**	-0.171	-0.313	0.219	0.550	-0.465	0.384	0.547	0.341	0.475	0.660	0.082	1					
Bio15	-0.405	0.665	-0.031	0.449	-0.017	-0.541	0.529	-0.026	-0.519	-0.094	-0.481	-0.502	0.318	-0.694	1				
Bio16	0.734	-0.402	0.402	-0.537	0.139	0.672	-0.616	0.438	0.685	0.355	0.698	**0.830**	**0.930**	0.239	0.047	1			
Bio17	0.515	**-0.807**	-0.158	-0.322	0.217	0.557	-0.473	0.383	0.555	0.342	0.484	0.679	0.095	**0.999**	-0.700	0.258	1		
Bio18	0.686	-0.374	0.397	-0.514	0.097	0.628	-0.588	0.411	0.646	0.313	0.656	**0.808**	**0.937**	0.237	0.091	**0.995**	0.254	1	
Bio19	0.503	-0.797	-0.165	-0.312	0.216	0.545	-0.461	0.379	0.545	0.338	0.472	0.668	0.087	**0.999**	-0.693	0.248	**1**	0.244	1

Notice: results with $r > 0.8$ were marked by bold fonts.
